# Effects of a Flavonoid-Rich Fraction on the Acquisition and Extinction of Fear Memory: Pharmacological and Molecular Approaches

**DOI:** 10.3389/fnbeh.2015.00345

**Published:** 2016-01-05

**Authors:** Daniela R. de Oliveira, Claudia R. Zamberlam, Gizelda M. Rêgo, Alberto Cavalheiro, Janete M. Cerutti, Suzete M. Cerutti

**Affiliations:** ^1^Cellular and Behavioral Pharmacology Laboratory, Department of Biological Science, Universidade Federal de São PauloSão Paulo, Brazil; ^2^Genetic Bases of Thyroid Tumor Laboratory, Division of Genetics, Department of Morphology and Genetics, Universidade Federal de São PauloSão Paulo, Brazil; ^3^Department of Forestry Colombo, Brazilian Agricultural Research CorporationColombo, Brazil; ^4^Institute of Chemistry, Nuclei of Bioassay, Biosynthesis and Ecophysiology of Natural Products, São Paulo State University, Universidade Estadual PaulistaAraraquara, Brazil

**Keywords:** flavones, fear memory, GABA_A_R, 5-HT_1A_R, GluN2B-NMDAR

## Abstract

The effects of flavonoids have been correlated with their ability to modulate the glutamatergic, serotoninergic, and GABAergic neurotransmission; the major targets of these substances are N-methyl-D-aspartic acid receptor (NMDARs), serotonin type1A receptor (5-HT_1A_Rs), and the gamma-aminobutyric acid type A receptors (GABA_A_Rs). Several studies showed that these receptors are involved in the acquisition and extinction of fear memory. This study assessed the effects of treatment prior to conditioning with a flavonoid-rich fraction from the stem bark of *Erythrina falcata* (FfB) on the acquisition and extinction of the conditioned suppression following pharmacological manipulations and on gene expression in the dorsal hippocampus (DH). Adult male *Wistar* rats were treated before conditioned fear with FfB, vehicle, an agonist or antagonist of the 5-HT_1A_R, GABA_A_Rs or the GluN2B-NMDAR or one of these antagonists before FfB treatment. The effects of these treatments on fear memory retrieval, extinction training and extinction retrieval were evaluated at 48, 72, and 98 h after conditioning, respectively. We found that activation of GABA_A_Rs and inactivation of GluN2B-NMDARs play important roles in the acquisition of lick response suppression. FfB reversed the effect of blocking GluN2B-NMDARs on the conditioned fear and induced the spontaneous recovery. Blocking the 5-HT_1A_R and the GluN2B-NMDAR before FfB treatment seemed to be associated with weakening of the spontaneous recovery. Expression of analysis of DH samples via qPCR showed that FfB treatment resulted in the overexpression of *Htr1a, Grin2a, Gabra5*, and *Erk2* after the retention test and of *Htr1a* and *Erk2* after the extinction retention test. Moreover, blocking the 5-HT1ARs and the GluN2B-NMDARs before FfB treatment resulted in reduced *Htr1a* and *Grin2b* expression after the retention test, but played a distinct role *in Grin2a* and *Erk2* expression, according session evaluated. We show for the first time that the serotoninergic and glutamatergic receptors are important targets for the effect of FfB on the conditioned fear and spontaneous recovery, in which the ERK signaling pathway appears to be modulated. Further, these results provide important information regarding the role of the DH in conditioned suppression. Taken together, our data suggest that FfB represents a potential therapy for preventing or treating memory impairments.

## Introduction

Several studies have investigated the effects of the extracts of flavonoid-rich plants or flavonoid molecules as potent modulators of brain structure and function, including their neuroprotective and chemopreventive properties and their beneficial effects on memory and cognition. The effects of flavonoids have been correlated with their ability to modulate the phosphorylation state of intracellular proteins via the activation or inhibition of protein kinases and phosphatases (Gamet-Payrastre et al., 1999; Wang et al., 1999; Schroeter et al., 2002; Li et al., 2003; Hoffman et al., 2004; Joseph et al., 2005; Maher et al., 2006; Nakajima et al., 2007; Spencer, 2007; Vauzour et al., 2008; Williams et al., 2008; Lovera et al., 2012; Mansuri et al., 2014), to increase the level of 5-HT and its metabolites (Zhang et al., 2012) or to alter expression of GABA_A_ receptors (GABA_A_Rs) and/or glutamatergic N-methyl-D-aspartic acid (NMDA) receptors (NMDARs) (Wang et al., 2005, 2008; Rendeiro et al., 2014). In addition, studies addressing the effects of specific flavonoid subgroups, including flavanols, anthocyanins, flavanones, and flavones, have shown that these constituents display potential to act as cognition-enhancing and neuroprotective agents (Vauzour et al., 2008; Kehr et al., 2012; Rendeiro et al., 2013, 2014; Vauzour, 2014), to prevent many forms of cerebrovascular disease, or to function as anti-anxiety drugs (Hasenöhrl et al., 1998; Spencer, 2008; Zhang et al., 2012). Although studies *ex vivo, in vivo*, and *in vitro* have provided evidence supporting the effects of flavonoids on the central nervous system, the cellular, and molecular pathways through which these compounds modulate memory formation are not completely elucidated (Youdim et al., 2004; Nakajima et al., 2007; Spencer, 2007; Williams et al., 2008; Ballesteros et al., 2014; Kimura et al., 2014; Rendeiro et al., 2014). However, several studies have established that the hippocampus, which plays a central role as a substrate of fear memory and anxiety (Fendt and Fanselow, 1999; Sanders et al., 2003) and which is a component of the Behavioral Inhibition System (McNaughton and Gray, 2000), appears to be a target for the mnemonic effects of flavonoid metabolites (Bannerman et al., 2004; Wang et al., 2006, 2009; Williams et al., 2008; Rendeiro et al., 2012, 2014; Oliveira et al., 2013; Vauzour, 2014).

Previous studies from our laboratory have demonstrated the role of flavonoid-rich plant extracts, such as a standardized extract of *Ginkgo biloba* L. (EGb), in the modulation of fear memory (Oliveira et al., 2009, 2013) by inducing differential CREB-1, GAP-43, and GFAP gene and protein expression in the dorsal hippocampus (DH), the prefrontal cortex and the amygdaloid complex. Further, we have established that crude extracts, fractions, and flavonoid molecules isolated from the stem bark of *Erythrina falcata* (CE) improved the acquisition of conditioned fear as evaluated by single-trial, step-down inhibitory avoidance (IA) (de Oliveira et al., 2014). Additionally, we used an IA procedure to show for the first time that treatment with flavones produces another well-established conditioning phenomenon, spontaneous recovery (de Oliveira et al., 2014). These findings corroborate with the results described in the literature and expand the understanding that flavonoids act as cognition-enhancing agents. However, these results raise new questions, which are highlighted below.

The first question concerns the anti-anxiety properties and cognitive effects of the flavonoid-rich fraction from CE, given the various actions of flavonoids on the central nervous system. Despite the close relationship between fear memory and anxiety, these functions are dissociable at the behavioral, pharmacological, molecular, and neuroanatomical levels (McNaughton and Corr, 2004; Kalueff, 2007; Nakajima et al., 2007). The conditioned emotional response (CER) is a suitable animal model for studying the behavioral, pharmacological, and molecular mechanisms underlying fear memory and anxiety. To assess these phenomena, our lab has used the conditioned suppression of the lick response, in which the conditional stimulus (CS, tone), when associated with a noxious unconditioned stimulus (US, footshock), ultimately suppresses the licking response reinforced by water; i.e., the CS leads to the suppression of the ongoing behavior (Blanchard and Blanchard, 1969; Bolles and Collier, 1976; Fanselow, 1980; Sotty et al., 1996; Sanders et al., 2003). Fear responses (flight/fight/freezing) increase systematically as fear memory is acquired and decrease as fear memory is extinguished (Sotty et al., 1996; Liu et al., 2004; Apergis-Schoute et al., 2005; Davis, 2006; Erlich et al., 2012; Furini et al., 2014). Conditioned fear responses are insensitive to anxiolytic drugs (McNaughton and Corr, 2004), but several works show that treatment with diazepam, an anxiolytic drug that is widely used in the clinic, prior to the conditioning session disrupts the initial acquisition of learned fear (Jensen et al., 1979; Izquierdo and Medina, 1991; Makkar et al., 2010), decreases the occurrence of freezing responses in a dose-dependent manner in rats (Fanselow and Helmstetter, 1988; Decker et al., 1990; Beck and Fibiger, 1995; Malkani and Rosen, 2000; Isoardi et al., 2004; Yeh et al., 2015) and impairs the acquisition of conditioned suppression (Oliveira et al., 2009). Anxiolytic compounds were effective in reducing the inhibitory response of animals to an aversive stimulus, which alleviated the suppression of the CER (McNaughton and Gray, 2000; Miyamoto et al., 2000; George et al., 2009). McNaughton and col. showed that anxiolytic drugs reduced theta frequency in the hippocampus (Coop et al., 1991; Munn and McNaughton, 2008). In this sense, the sensitivity of the CER to anxiolytic drugs, such as benzodiazepines and agonists of 5-HT_1A_ receptors (5-HT_1A_Rs) (Millenson and Leslie, 1974; Davis, 1990; Stanhope and Dourish, 1996; Mirza et al., 2005; George et al., 2009; Oliveira et al., 2009), substantiates the use of this model to investigate the fundamental mechanisms underlying the effects of anti-anxiety drugs in addition to their function in alleviating conditioned fear in rodents.

The second question concerns the neurochemical mechanisms underlying both the acquisition and the extinction of conditioned suppression, as well as the role of the flavonoid-rich fraction from the stem bark of *Erythrina falcata* (FfB) in modulating these processes. We primarily focused on the molecular events underlying the acquisition of fear memory and the modulatory effects of FfB. Further, we were interested in determining whether treatment with flavonoids prior to conditioning can modulate the extinction process. Studies demonstrating the involvement of glutamatergic, serotoninergic and GABAergic neurotransmission in the acquisition of fear memory have been accumulating in past decades; the major targets of these neurotransmitters are NMDARs, 5-HT_1A_Rs, and GABA_A_Rs, respectively (Santini et al., 2001; Davis and Myers, 2002; Lin et al., 2003; Quirk and Mueller, 2008; Kim and Richardson, 2010), and the modulation of these receptors in the hippocampus is essential for the acquisition and consolidation of fear memory (Izquierdo, 1997; Cammarota et al., 2000; Alonso et al., 2002; Milad et al., 2007). Similarly, these changes are essential to consolidation of fear extinction (Myers and Davis, 2002). These effects are mediated by the activity of kinases and phosphatases, and ERK1/2 activation has been described to be involved in several cellular changes associated with long-term memory (LTM) (Atkins et al., 1998; Cammarota et al., 2000). Blocking NMDARs in the prefrontal cortex and the hippocampus is known to result in a deficit in the acquisition of fear extinction (Lissek and Güntürkün, 2003) and the retrieval of fear extinction (Lengersdorf et al., 2014). Evidence from *in vitro* and *in vivo* studies showed that flavones modulate GABA_A_Rs and GluN2B-NMDARs, but few studies have been conducted on the mechanisms underlying the modulatory effects of flavonoids on these processes. Therefore, in our study, we sought to elucidate the neurochemical systems involved in the acquisition of fear memory in the presence or absence of FfB treatment and to determine whether FfB treatment prior to conditioning modulates the extinction of fear memory. Further, we evaluated how these changes may control or be controlled by the activation or inhibition of specific receptors using pharmacological agonists or antagonists.

Therefore, the contributions of the glutamatergic, serotoninergic, and GABAergic systems, as well as the interactions between these systems, to the effects of FfB on the acquisition and extinction of conditioned suppression were assessed for the first time by administering agonists, antagonists of receptors for GABA, glutamate (NMDA) and 5-HT or one these antagonists before FfB prior the conditioning session. Additionally, we evaluated the mRNA expression levels of the GluN2A and GluN2B subunits of the NMDAR, the receptor subunits GABA_A_R and 5-HT_1A_R and ERK1/2 in the DH of controls and treated rats subjected to acquisition and extinction of conditioned fear.

This combination of molecular, behavioral and pharmacological analyses advances our understanding of the role of flavones in fear memory and anxiety. The findings regarding the molecular mechanisms of flavone action appear to be promising with respect to the development of new therapeutic strategies for the treatment of cognitive deficits or anxiety disorders. Moreover, we assessed the contribution of the hippocampus to these processes. In particular, we focused on the suppression of the licking response as a behavioral model and the hippocampus as a key component of the neural circuitry involved in the acquisition, consolidation and extinction of fear memory in animals and humans, as the hippocampus may represent a target for the action of FfB.

## Experimental procedures

### Drugs and reagents

Methanol (HPLC grade) was obtained from Merck (Darmstadt, Germany). Formic acid, ethanol, *n*-butanol, and Tween®-80 were obtained from Synth (Diadema, Brazil). Vitexin and isovitexin standards (99.99%) were purchased from Sigma-Aldrich (São Paulo, Brazil). The 6-C-glycoside-diosmetin and vicenin-2 standards were generated in our laboratory according to the methods described by de Oliveira et al. (2014). Valium® (diazepam) was purchased from Roche (São Paulo, Brazil). Sintocalmy® (standardized extract of *Passiflora incarnate* L.—extract ACH 06) was obtained from Aché (Guarulhos, Brazil). Ro25-6981, picrotoxin and (S)-WAY100135 were purchased from Tocris Biosciences (Ellisville, MO, USA). NMDA was obtained from Sigma-Aldrich (São Paulo, Brazil). Buspirone hydrochloride was obtained from LIBBS Pharmaceutical Ltd (São Paulo, Brazil).

#### Standardized FfB preparation

FfB was obtained by flash chromatography, as previously described by de Oliveira et al. (2014). Additionally, the FfB was analyzed using high-performance liquid chromatography (HPLC) combined with electrospray ionization tandem mass spectrometry (HPLC-ESI/MS^n^) using a Thermo LCQ Fleet System mass spectrometer (Thermo Scientific, San Diego, CA, USA) equipped with an electrospray interface (ESI) and an HPLC (model Accela, Thermo Scientific). FfB separation was performed using a Luna® C18 column (250 × 4.60 mm; Phenomenex, Torrance, CA, USA) at room temperature. The mobile phase consisted of 0.1% aqueous formic acid-water (A) and methanol (B). A gradient elution method of A/B (from 64:36 to 1:1, v/v) was applied over 50 min. Ultraviolet (DAD) detection was performed at 330 nm; the flow rate was maintained at 0.8 mL/min; the sample concentrations were 1 mg.mL^−1^; and the injection volume was 10 μL. The column effluents were analyzed by ESI-MS in negative ion mode in the mass-to-charge ratio (*m/z)* range of 50–2000, with a scan time of 0.3 s in the centroid mode. The ESI conditions were as follows: nebulizer gas (nitrogen), 30 psi; drying gas, 60 L.min^−1^; drying temperature, 280°C; capillary voltage, 4000 V; collision gas, nitrogen; and collision energy,1 V. The data were acquired in the MS and MS^n^ scanning modes. The CE was dissolved in H_2_O: MeOH (1:1v/v) and was infused directly via a syringe pump (flow rate 5 μL.min^−1^) in the ESI source. The data were analyzed using Xcalibur 2.0 Software® (Thermo Scientific).

The flavonoids present in the FfB were quantified by HPLC-DAD using a Luna® C18 column (Phenomenex, Torrance, CA, USA; 250 mm × 4.60 mm, 5 μm). The mobile phase consisted of 0.1% aqueous formic acid (A) and methanol (B). An isocratic elution method of A/B (64:36, v/v) was applied for 50 min. UV spectra were recorded from 200 to 400 nm, and the chromatogram was monitored at 254, 280, and 330 nm. The flow rate was maintained at 1 mL.min^−1^; the sample concentration was 1 mg.mL^−1^; and the injection volume was 20 μL. Analytical curves were obtained for vitexin, isovitexin, vicenin-2, and 6-C-glycoside-diosmetin (1 mg.mL^−1^ of each compound in 80:20 methanol/water), which peaked at concentrations ranging from 100 to 1.000 mg.mL^−1^. The sample peak areas were integrated at 254 nm. All of the procedures were performed in triplicate.

### Behavioral and pharmacological effects of acute treatment with FfB before conditioning on the acquisition and extinction of conditioned suppression

#### Subjects

A total of 470 adult male *Wistar* rats (±250–300 g) were obtained from the Center for the Development of Experimental Medicine and Biology (CEDEME, Federal University of Sao Paulo, SP, Brazil). The rats were housed 5 animals/cage. For 15 days, the animals had free access to food and water under a 12 h:12 h dark:light cycle (lights on at 6:00–18:00 h) at a controlled temperature (21°C ± 2°C) and relative humidity (53 ± 2%). These conditions were maintained throughout the experimental period. One minute prior to the experimental sessions, each rat was placed in an individual cage for transportation to the testing room. All of the procedures for manipulation of the animals were consistent with the Ethical Principles in Animal Research adopted by the Brazilian College for Animal Experimentation (COBEA) and were performed as suggested by the APA Guidelines for Ethical Conduct in the Care and Use of Animals. The protocol was approved by the Committee on the Ethics of Animal Experiments of the Federal University of Sao Paulo (Permit Number: 840560). After completion of the behavioral experiments, the animals were decapitated, and the DH was extracted within 40–60 s using a magnifying glass, immediately frozen on dry ice, and maintained at −80°C until gene expression analysis. All behavioral procedures were conduced during the light phase of the dark:light cycle, and all efforts were made to minimize suffering.

#### Systemic administration

Diazepam and buspirone hydrochloride (a GABA_A_R and a 5-HT_1A_R agonist, respectively), Sintocalmy®(a standardized extract of *Passiflora incarnata* L.-extract ACH 06, containing 7% (21 mg) total flavonoids expressed as vitexin) and three different concentrations of FfB were dissolved in 12% Tween®-80 and administered orally via intragastric gavage (IG) 30 min before each conditioning session. The GABA_A_R, 5-HT_1A_R, and GluN2B-NMDAR antagonists (picrotoxin, S-WAY 100135 and Ro25-6981, respectively) and NMDA (an NMDAR agonist) were dissolved in saline and injected intraperitoneally (i.p.) 20 min before each conditioning session or prior to treatment with FfB. When an antagonist was administered before with FfB, the drugs were administered −50 or −30 min before conditioning, respectively. No drugs were administered before the retention test, extinction training or the extinction retention test. The drugs were administered i.p. or IG in a volume of 1 mL. The doses, administration routes and vehicles used to dissolve of the antagonists and agonists were chosen based on previous reports (Aguilar et al., 1997; Risbrough et al., 2003; Oliveira et al., 2009).

### Experimental procedure

Rats were randomly assigned to the control group or the FfB group (*n* = 20 per subgroup) (Table 1). The control group was subdivided into 12 subgroups as follows: (i) the paired stimulus conditioned/unconditioned stimulus (CS-US) subgroup; (ii) the unconditioned subgroup [no footshock, i.e., only tone (CS); as such, these animals were used as controls for learning]; (iii–iv) the negative control subgroups (12% Tween®-80 or saline); (v–xi) the positive control subgroups (4.0 mg.Kg^−1^ diazepam; 10.0 mg.Kg^−1^ NMDA; 10.0 mg.Kg^−1^ buspirone hydrochloride; 600 mg.Kg^−1^ Sintocalmy®; 0.75 mg.Kg^−1^ picrotoxin; 3.0 mg.Kg^−1^ Ro25-6981; or 0.3 mg.Kg^−1^ (S)-WAY 100135; these animals were used as controls for treatment with the respective drug together with FfB); and (xvii) a naïve subgroup (*n* = 10), which was used as a control for gene expression. The FfB groups were also divided into 12 subgroups as follows: (xiii–xiv) FfB alone (0.15 mg.Kg^−1^ FfB, 0.30 mg.Kg^−1^ FfB or 0.65 mg.Kg^−1^ FfB); (xv–xvii) picrotoxin+ FfB (Picro+0.15 mg.Kg^−1^ FfB; Picro+0.30 mg.Kg^−1^FfB; or Picro+0.65 mg.Kg^−1^ FfB); (xviii–xx) Ro25-6981+ FfB (Ro+0.15 mg.Kg^−1^ FfB; Ro+0.30 mg.Kg^−1^ FfB or Ro+0.65 mg.Kg^−1^ FfB); and (xxi–xxiii) (S)-WAY+ FfB [(S)-WAY+ 0.15 mg.Kg^−1^ FfB; (S)-WAY+0.30 mg.Kg^−1^ FfB or (S)-WAY+0.65 mg.Kg^−1^ FfB]. Half of the rats (*n* = 10/subgroup) were sacrificed after the retention test ended. The remaining half (*n* = 10/subgroup) were subjected to extinction training and an extinction retention test of the CER and were sacrificed 3 h after the conclusion of the extinction retention test.

**Table 1:**
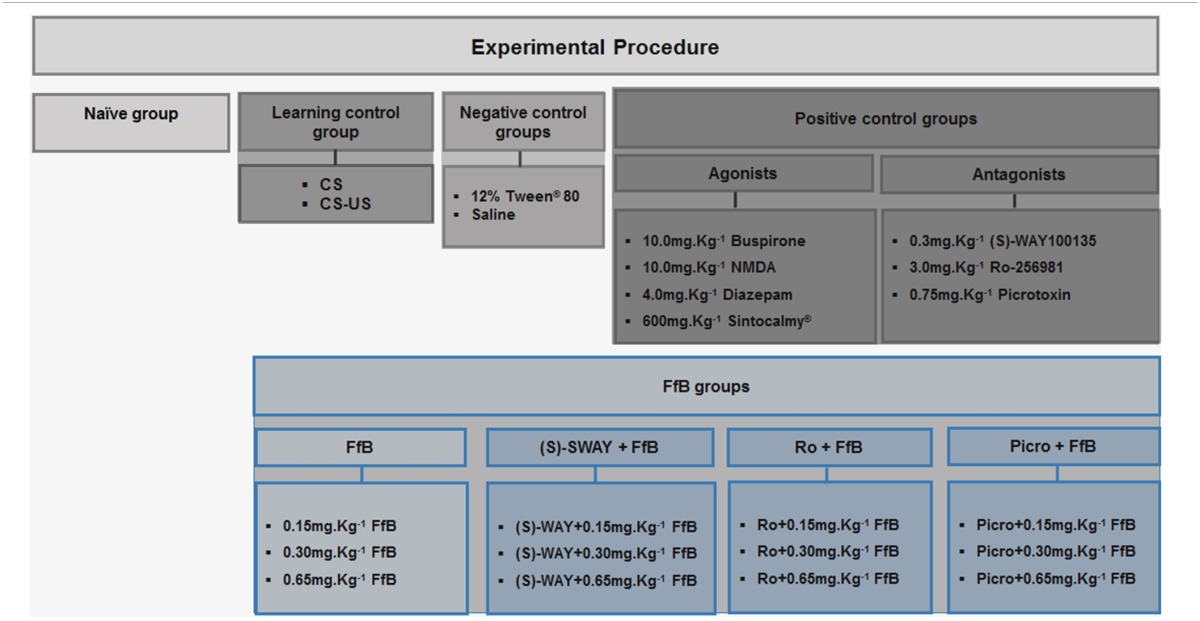
**Experimental groups**.

#### Behavioral apparatus

Rats were fear conditioned in a lick-operant chamber. Briefly, the experimental chambers consisted of an aluminum (side walls) and Plexiglas (ceiling and hinged front door) box measuring 25 × 25 × 20 cm set inside a sound-attenuation cabinet (53 × 65 × 50 cm). Three identical chambers and cabinets were used in all experiments. The floor consisted of stainless steel rods connected to grid shockers (model EP 107R, Insight, Ribeirão Petro, Brazil) set to deliver 0.4 mA, 0.5 s scrambled shocks, which were used as the US. A speaker positioned on top of the square, which produced a 2 kHz, 85 dB sound for 30 s, was used as the CS. A licking spout was slipped into the cage through a hole in the middle of the wall of the chamber; this hole protruded from the lateral wall 5.0 cm above the grid floor. Stimulus presentation and data recording were controlled using software (Refor II Software®, Insight) and a central controller box (Insight). The chambers were cleaned with 10% ethanol before each test.

#### Behavioral procedure

**Figure 1:**
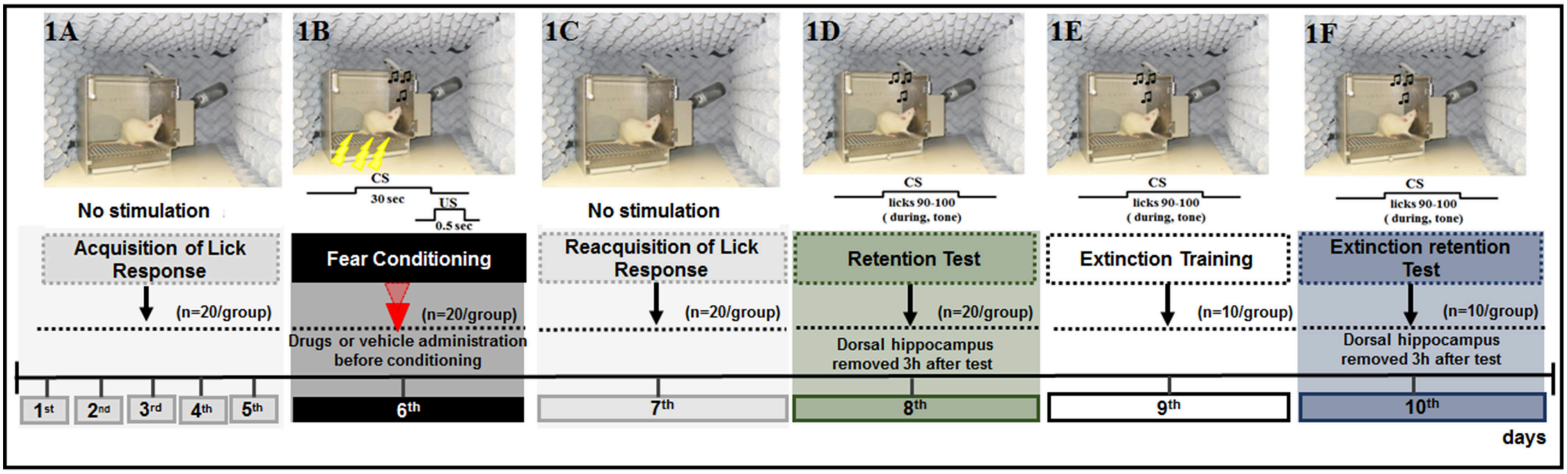
**Schematic outline of the experimental procedure and drug administration time, common to all animals, except for the CS-US, and CS groups, in which did not receive the drug or vehicle**. **(1A)** The animals were submitted to acquisition of the licking response for 5 days (baseline behavior). **(1B)** On day 6, the animals were submitted to four associations of CS-US (conditioning). **(1C)** Twenty-four hours later (day 7), the animals were submitted to re-acquisition of licking behavior, in conditions identical to those of the acquisition period (1–5 days). Retention Test **(1D)**, Extinction Training **(1E)**, and Extinction Test **(1F)** were performed on days 8, 9, and 10, respectively. Ten CS trials were presented at these times. No drugs were administered during the tests and extinction training sessions.

The behavioral procedure was conducted for 8 or 10 days, according to the experimental design, to assess the acquisition or extinction of a CER, respectively. All rats, except for those in the CS and naïve subgroups, were subjected to a procedure to induce acquisition of fear memory (*n* = 20/group) (Figure 1). Three hours after the completion of the fear acquisition test (8^th^ day), half of the rats were decapitated. Then, the DH was extracted within 40–60 s using a magnifying glass, immediately frozen on dry ice, and maintained at −80°C until gene expression analysis (acquisition analysis) (*n* = 3/subgroup). The remaining half of the animals (*n* = 10/subgroup) were subjected to extinction training (9^th^ day) and an extinction retention test (10^th^ day) performed on each of the two consecutive days following the acquisition test. Three hours after completing the extinction retention test, these rats were decapitated, and the DH was extracted as described above (*n* = 3/group).

##### Suppression of the licking response

The animals were deprived of water on a daily basis for 12–16 h before all experimental sessions. For five consecutive days, the rats were placed individually in the chamber once a day for 20 min sessions with free access to the drinking spout to obtain a stable baseline of drinking behavior, but no other stimuli were presented (Figure 1A). After the administration of drugs or vehicle, each rat was gently placed in the experimental chamber, and after 5 min, the animal was submitted to four tone-shock (CS-US) pairings (fear conditioning, 6^th^ day; Figure 1B). Twenty-four hours after fear conditioning, the animals were subjected to reacquisition of the licking response sessions (7^th^ day) as performed during the acquisition of the licking response to re-establish drinking behavior after conditioning and to reduce contextual cues (Figure 1C). The retention test was performed 48 h after acquisition (8^th^ day) to evaluate the acquisition of fear memory as well as the effects of drug treatment. Here, each rat was placed in the experimental chamber with free access to the water spout and was subjected to the CS on 10 consecutive trials, in which the time to complete 10 licks pre-tone (no CS) and during the tone (CS) were recorded, and the suppression ratio (SR) was calculated for each trial. The tone was presented immediately after the animal completed its 90^th^ lick and was switched off after its 100^th^ lick (Figure 1D). The latency to complete licks 0–80 was recorded to ensure that the rats were licking when the tone was presented, but this value was not used to calculate the suppression of the lick response. The latency to complete licks 81–90 was measured as a control for time in the absence of a tone and was used to calculate the SR.

Therefore, the SR was calculated as the ratio of B/(A+B) for each rat, where A is the time to complete 10 licks pre-tone (pre-CS), i.e., time to complete licks 81–90 and B is the time to complete 10 licks during the CS, i.e., time to complete licks 91–100.

##### Extinction of suppression of the licking response

Analysis of the effects of FfB on extinction was performed using the behavioral protocol described for acquisition. All rats were subjected to tests of adaptation (1^st^–5^th^ days), acquisition (6^th^ day), reacquisition (7^th^ day), and retention of the CER (8^th^ day). Seventy-two hours (9^th^ day) and ninety-six hours (10^th^ day) after fear conditioning, the rats were placed in the experimental chamber for extinction training and extinction test sessions, respectively (Figures 1E,F). In both sessions, the latencies to complete licks before the tone and during the tone for 10 consecutive CS presentations were recorded as described for the 8^th^ day.

#### Data analysis

The data from the first CS presentation indicated whether the association was learned. An SR approaching 1.0 indicates total suppression (high fear), whereas an SR ≤ 0.5 indicates no suppression (low fear), i.e., failure to learn the tone-shock relationship. The data are reported as the means ±SEM. A Two-way analysis of variance (ANOVA) was used to test for the presence of the effects of group and trial and the interaction between these variables; two fixed factors (group and trial), one random factor (rat), and repeated measurement of the trials were considered. *P* < 0.05 were considered significant. Graph Pad 6.0 Software® (version 6.0; Graph Pad Inc., San Diego, CA, USA) was used for data analysis.

#### Expression of *Gabra5, Htr1a, Grin2a, Grin2b*, and *Mapk1/Erk2* by quantitative PCR (qPCR) following treatments before conditioning and behavioral analysis

The analysis of gene expression in the DH samples was extracted 3 h after the completion of the retention test or the extinction retention test as previously described. The candidate genes gamma-aminobutyric acid receptor subunit alpha-5 (*Gabra5*), 5-hydroxytryptamine (serotonin) receptor, subunit 1A (*Htr1a*), glutamate receptor ionotropic, NMDAR subunit GluN2A (*Grin2a*), glutamate receptor ionotropic, NMDAR subunit GluN2B (*Grin2b*), and extracellular signal-regulated kinase 2 (*Erk2*) were investigated. To this end, total RNA was isolated using Trizol reagent (Invitrogen Corp., Carlsbad, CA, USA) according to the manufacturer's recommendations. One microgram of total RNA was subjected to DNA-free DNase treatment (AMBION, Austin, TX, USA) and reverse-transcribed into cDNA using the SuperScript® III Reverse Transcriptase kit (Invitrogen Corp.) together with oligo_12−18_ primer and 10 units of an RNase inhibitor (Invitrogen Corp.). Reverse transcriptase-negative samples were prepared for each individual reaction and were used as controls for assay contamination. Aliquots of 1 μL of cDNA were used in 12 μL reactions containing SYBR Green Master Mix (PE Applied Biosystems, Foster City, CA) and 3pM of each primer for the target genes and the reference gene (RS8) as described previously (Cerutti et al., 2004). The primer sequences are displayed in (Table S1). The qPCR reactions were performed in triplicate, and the threshold for each cycle (Ct) was obtained using Applied Biosystems software (Applied Biosystems) and averaged [standard deviation (SD) ≤ 1]. Relative expression (RE) levels were calculated using the 2^−△△^^CT^ method (ddCt formula) as described previously (Cerutti et al., 2004). The vehicle (12% Tween®-80 or saline) was used as a control.

The analyses were performed using Graph Pad 6.0 Software® (version 6.0; Graph Pad Inc., San Diego, CA, USA). For candidate gene expression analysis, normality of the data was verified using the Shapiro-Wilk normality test. One-way ANOVA followed by a *post-hoc* Bonferroni test was performed to evaluate the relationships between the expression levels of *Gabra5, Htr1a, Grin2a, Grin2b*, and *Erk2* across groups. *P* < 0.05 were considered significant.

## Results

### Identification of flavonoids in FfB

The spectroscopic and chromatographic data of the peaks (1–6) of the FfB are summarized in Figures S1A,B. The identities, fragmentation patterns and UV spectra were confirmed as follows: (1) vicenin-2:λmax = 334, 271 nm, [M-H]^−^ = *m/z* 593; (2) vicenin-1:λmax = 332, 271 nm, [M-H]^−^ = *m/z* 563; (3) vitexin:λmax = 269, 235 nm, [M-H]^−^ = *m/z* 431; (4) isovitexin:λmax = 335, 271 nm, [M-H]^−^ = *m/z* 431; (5) 6-C-glycoside-diosmetin:λmax = 342, 270 nm, [M-H]^−^ = *m/z* 461; and (6) apigenin:λmax = 305, 265 nm, [M-H]^−^ = *m/z* 269. These results were consistent with those previously reported by de Oliveira et al. (2014). The identification of 6-C-glycoside-diosmetin, vicenin-2, vitexin and isovitexin was supported by the co-injection of the standards and FfB. The flavones (1, 3, 4, and 5) found in the FfB were quantified by HPLC-DAD, and the concentrations contained in the FfB were 0.15 mg/g vicenin-2, 0.20 mg/g vitexin, 0.30 mg/g isovitexin, and 0.25 mg/g 6-C-glycoside-diosmetin.

Despite the evidence from our studies, few studies have examined the effects of a flavonoid fraction on fear memory. Further, previous data from our group suggest that the FfB may modulate different neurochemical systems.

### Behavioral, pharmacological, and molecular analysis

**Figure 2:**
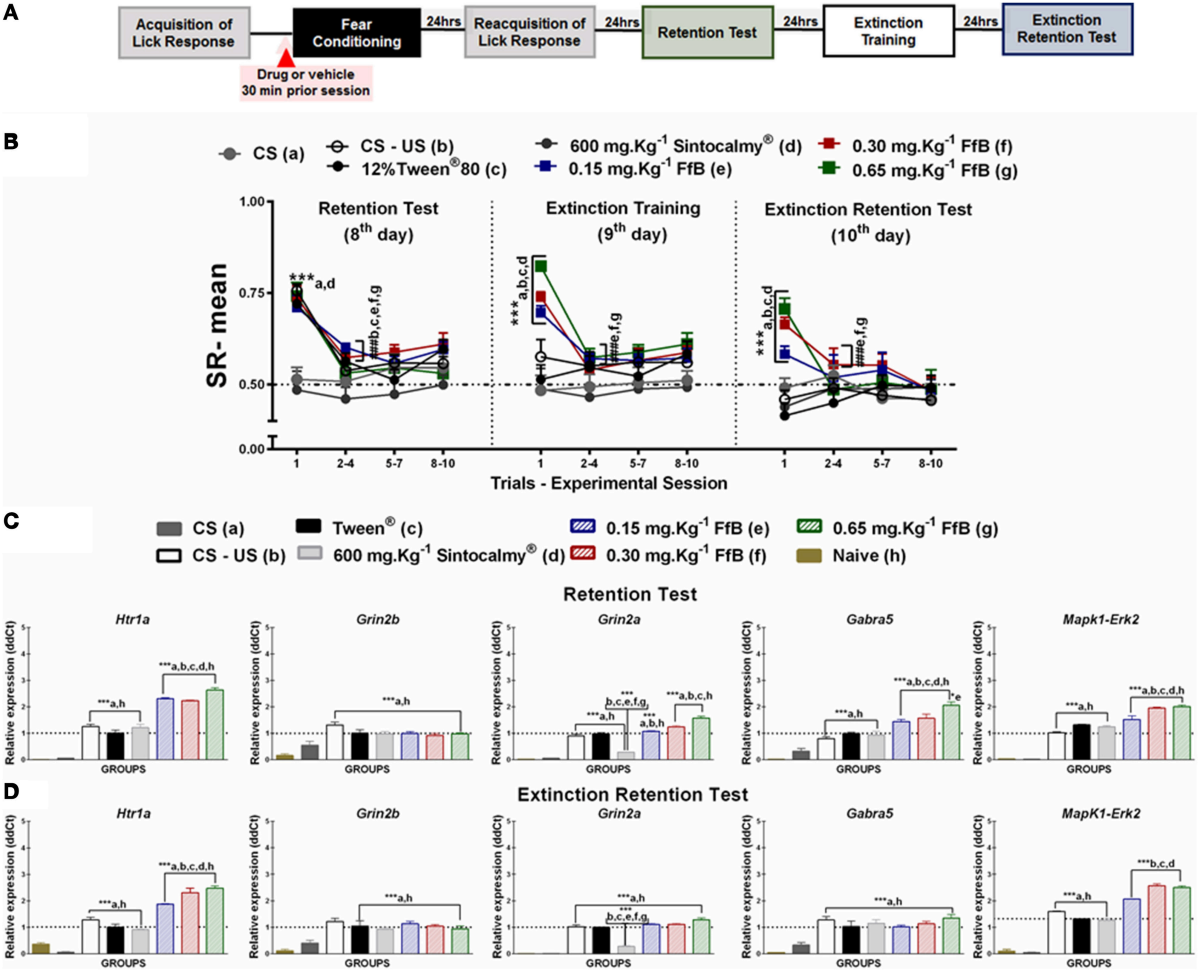
**(A)** Timeline illustrating the time points of drug administration and brain removal. **(B)** Mean SR of licking behavior in the retention test session (8^th^ day, *n* = 20/group), extinction training (9^th^ day, *n* = 10/group) and the extinction retention test (10^th^ day, *n* = 10/group). The first point indicates the mean SR for the CS, learning, Sintocalmy®, Tween®, 0.15 mg.Kg^−1^ FfB, 0.30 mg.Kg^−1^FfB, and 0.65 mg.Kg^−1^FfB subgroups. The subsequent data points represent the mean of nine trials in blocks of three trials. The drugs and vehicle were administered orally 30 min before the fear conditioning session; the CS and CS-US groups received no treatment. The data are reported as the means (±SEM). A repeated measures ANOVA was employed for the intra-group comparison of the retention test, extinction training and extinction retention test (CS presentation) results. This analysis was performed considering two fixed factors (group and trial) and one random factor (rat) using GraphPad Prism software. he relative *Htr1a, Grin2b, Grin2a, Gabra5*, and *Erk2* mRNA expression levels in the DH after acute treatment with Sintocalmy®, Tween®, 0.15 mg.Kg^−1^FfB, 0.30 mg.Kg^−1^FfB, or 0.65 mg.Kg^−1^FfB (*n* = 3/subgroup) followed by the retention test **(C)** or the extinction retention test **(D)**. The CS-US, CS, and naïve subgroups did not receive treatment (*n* = 3/subgroup). The values are expressed as the means (± SEM). ^*^P < 0.05 and ^***^P < 0.0001. ^###^*P* < 0.0001 according to ANOVA followed by *post-hoc* Bonferroni tests, when necessary.

**Figure 3:**
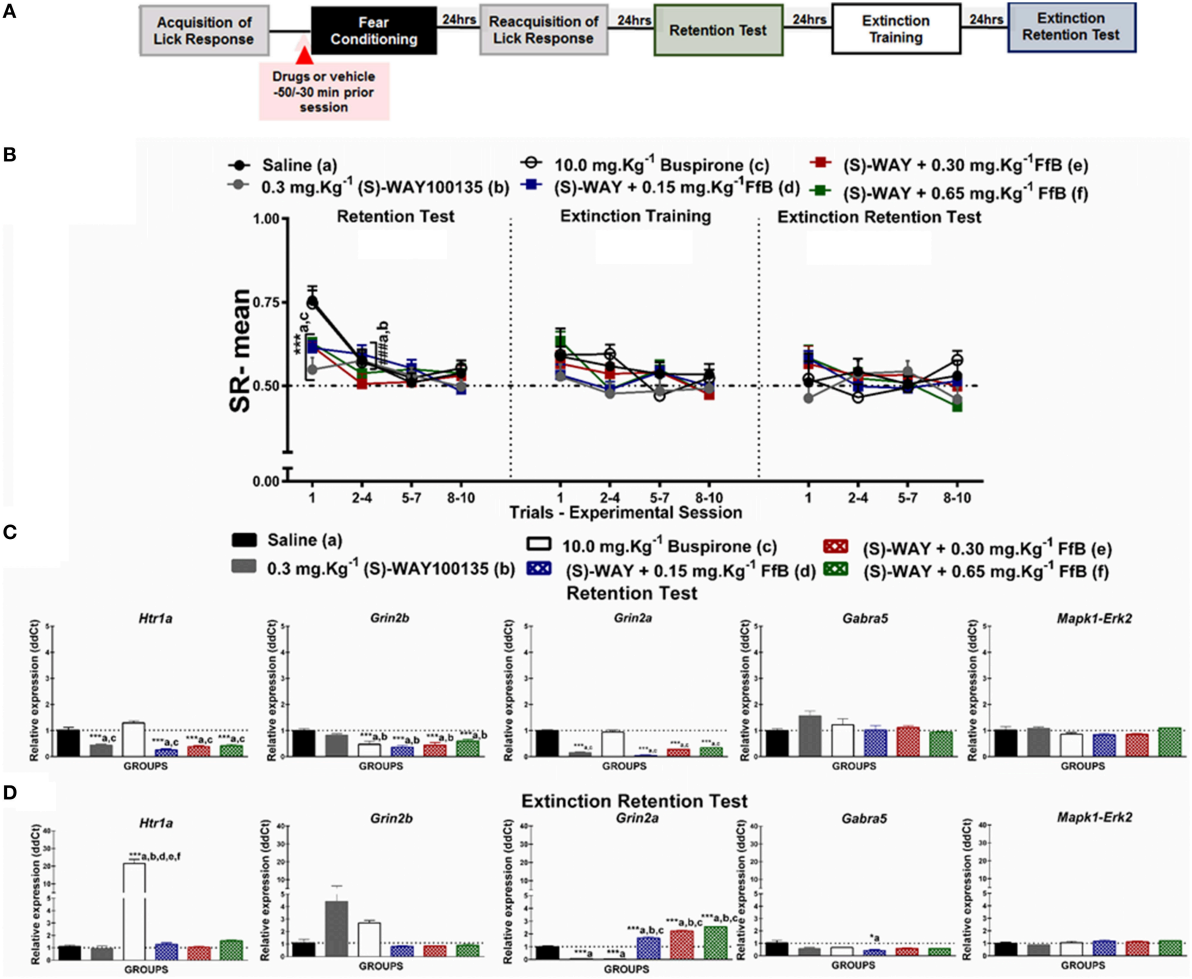
**(A)** Timeline illustrating the time points of drug administration and brain removal. **(B)** Mean SR of licking behavior in the test session (8^th^ day, *n* = 20/group), extinction training (9^th^ day, *n* = 10/group) and the extinction retention test (10^th^ day, *n* = 10/group). The first point indicates the mean SR for the saline, 0.3 mg.Kg^−1^ (S)-WAY100135, 10 mg.Kg^−1^ buspirone, and 0.3 mg.Kg^−1^ (S)-WAY100135+FfB (0.15 mg.Kg^−1^ FfB, 0.30 mg.Kg^−1^ FfB, or 0.65 mg.Kg^−1^ FfB) subgroups. The subsequent data points represent the mean of nine trials in blocks of three trials. The drugs and vehicle were administered orally 30 min before the fear conditioning session. The data are reported as the means (±SEM). A repeated measures ANOVA was employed for the intra-group comparison of the retention test, extinction training and extinction retention test (CS presentation) results. This analysis was performed considering two fixed factors (group and trial) and one random factor (rat) using GraphPad Prism software. The relative *Htr1a, Grin2b, Grin2a, Gabra5*, and *Erk2* mRNA expression levels in the DH after acute treatment with 0.3 mg.Kg^−1^ (S)-WAY100135, 10 mg.Kg^−1^ buspirone, 0.3 mg.Kg^−1^ (S)-WAY100135 + (FfB 0.15 mg.Kg^−1^ FfB, 0.30 mg.Kg^−1^ FfB, or 0.65 mg.Kg^−1^ FfB) or saline (*n* = 3/subgroup) followed by the retention test **(C)** orthe extinction retention test **(D)**. The values are expressed as the means (± SEM). ^*^P < 0.05 and ^***^P < 0.0001. ^###^*P* < 0.0001 according to ANOVA followed by *post-hoc* Bonferroni tests, when necessary.

**Figure 4:**
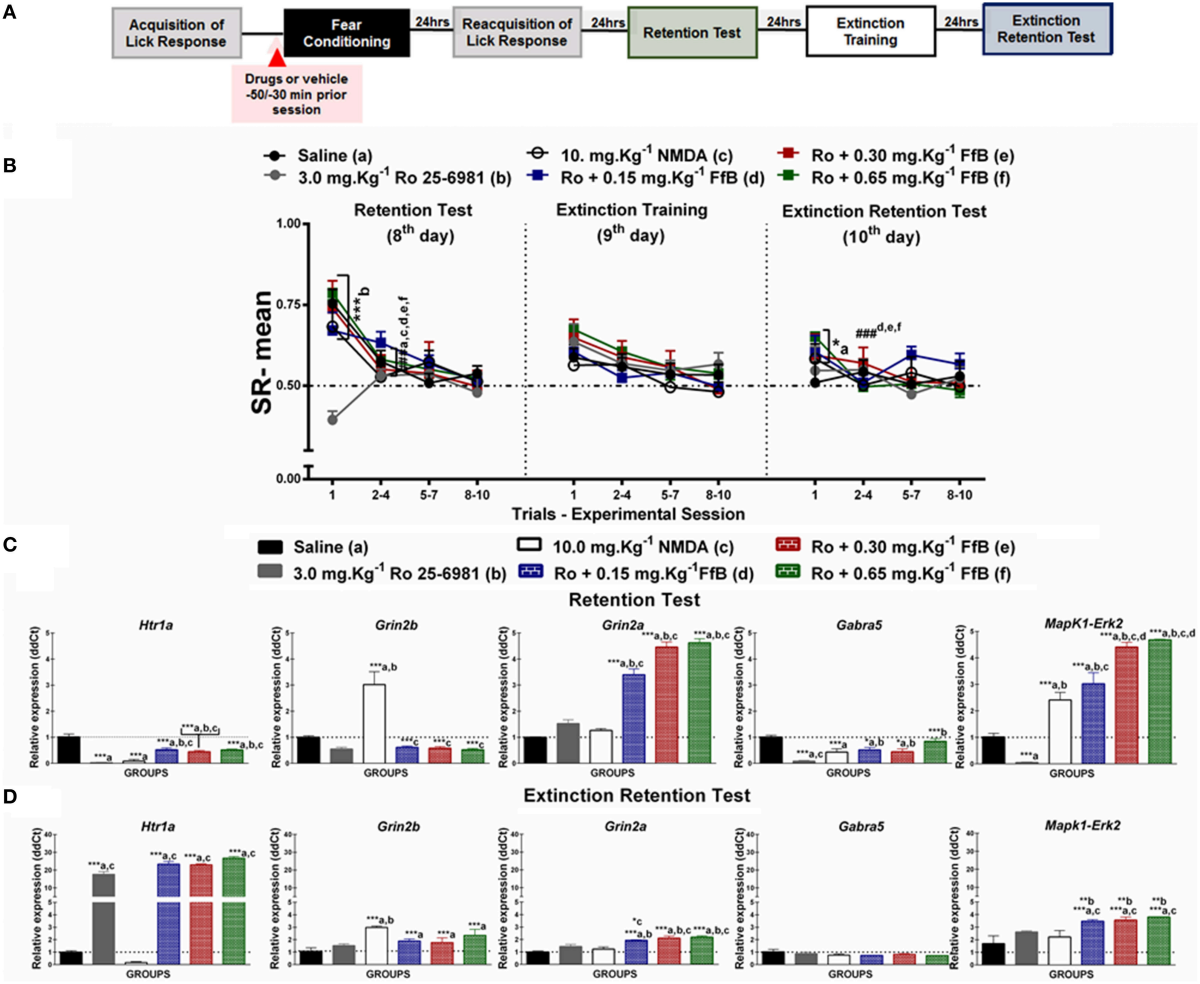
**(A)** Timeline illustrating the time points of drug administration and brain removal. **(B)** Mean SR of licking behavior in the test session (8^th^ day, *n* = 20/group), extinction training (9^th^ day, *n* = 10/group) and the extinction retention test (10^th^ day, *n* = 10/group). The first point indicates the mean SR for the 0.9% saline, 3.0 mg.Kg^−1^ Ro25-6981, 10 mg.Kg^−1^ NMDA, and Ro25-6981+FfB (0.15 mg.Kg^−1^ FfB, 0.30 mg.Kg^−1^ FfB, or 0.65 mg.Kg^−1^ FfB) subgroups. The subsequent data points represent the mean of nine trials in blocks of three trials. The drugs and vehicle were administered orally 30 min before the fear conditioning session. The data are reported as the means (±SEM). A repeated measures ANOVA was employed for the intra-group comparison of the retention test, extinction training and extinction retention test (CS presentation) results. This analysis was performed considering two fixed factors (group and trial) and one random factor (rat) using GraphPad Prism software. The relative *Htr1a, Grin2b, Grin2a, Gabra5*, and *Erk2* mRNA expression levels in the DH after acute treatment with 3.0 mg.Kg^−1^ Ro25-6981, 10.0 mg.Kg^−1^ NMDA, 3.0 mg.Kg^−1^ Ro25-6981+FfB (0.15 mg.Kg^−1^ FfB, 0.30 mg.Kg^−1^ FfB, or 0.65 mg.Kg^−1^ FfB), or saline (*n* = 3/subgroup) followed by the retention test **(C)** or the extinction retention test **(D)**. The values are expressed as the means (±SEM). ^*^P < 0.05, ^**^P < 0.01 and ^***^P < 0.0001. ^###^*P* < 0.0001 according to ANOVA followed by *post-hoc* Bonferroni tests, when necessary.

**Figure 5:**
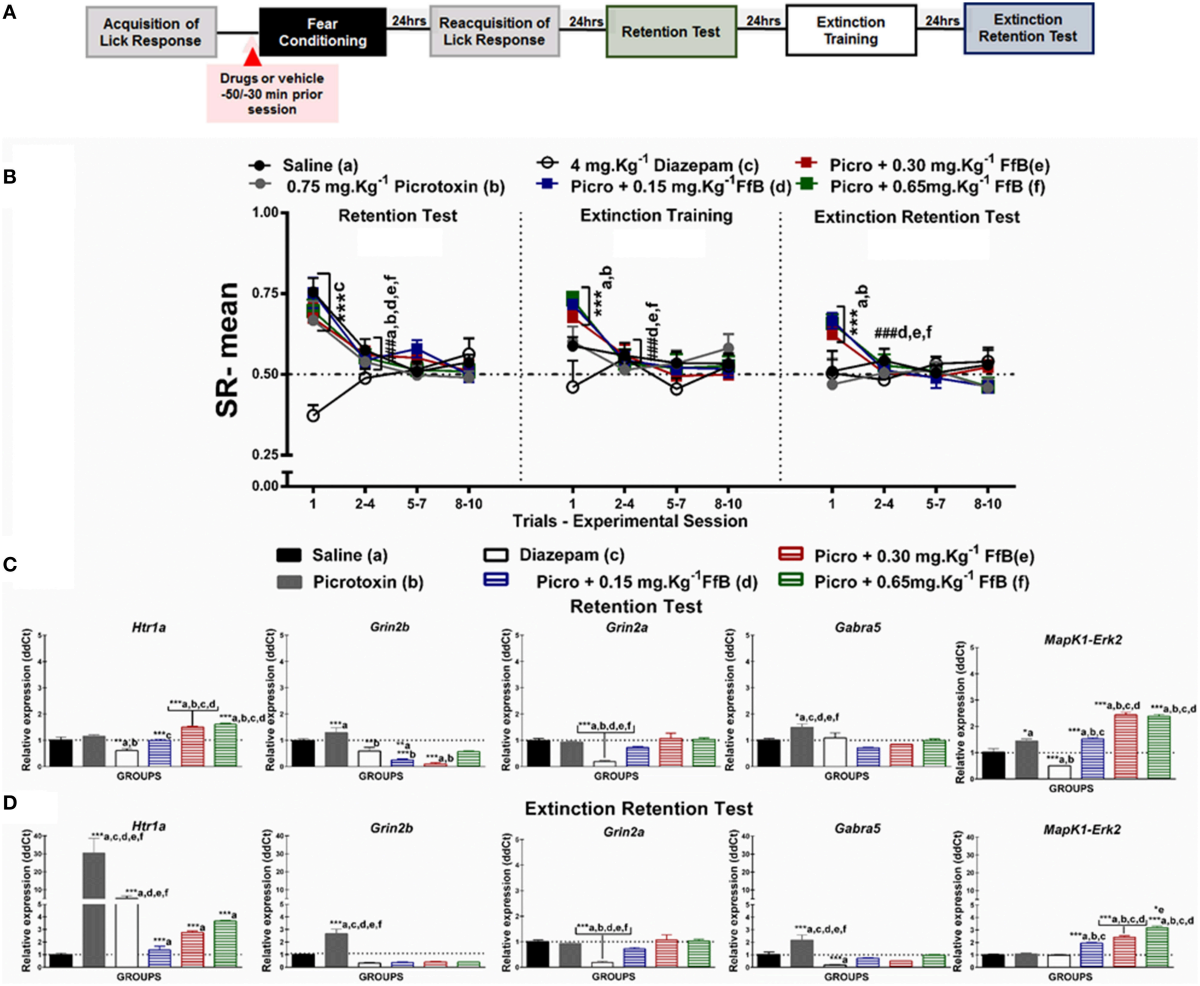
**(A)** Timeline illustrating the time points of drug administration and brain removal. **(B)** Mean SR of licking behavior in the test session (8^th^ day, *n* = 20/group), extinction training (9^th^ day, *n* = 10/group), and the extinction retention test (10^th^ day, *n* = 10/group). The first point indicates the mean SR of the 0.9% saline, 0.75 mg.Kg^−1^ picrotoxin, 4 mg.Kg^−1^ diazepam, and 0.75 mg.Kg^−1^ picrotoxin+FfB (0.15 mg.Kg^−1^ FfB, 0.30 mg.Kg^−1^ FfB, or 0.65 mg.Kg^−1^ FfB) subgroups. The subsequent data points represent the mean of nine trials in blocks of three trials. The drugs and vehicle were administered orally 30 min before the fear conditioning session. The data are reported as the means (±SEM). A repeated measures ANOVA was employed for the intra-group comparison of the retention test, extinction training and extinction retention test (CS presentation) results. This analysis was performed considering two fixed factors (group and trial) and one random factor (rat) using GraphPad Prism software. The relative *Htr1a, Grin2b, Grin2a, Gabra5*, and *Erk2* mRNA expression levels in the DH after acute treatment with 0.75 mg.Kg^−1^ picrotoxin, 4 mg.Kg^−1^ diazepam, 0.75 mg.Kg^−1^ picrotoxin+FfB (0.15 mg.Kg^−1^ FfB, 0.30 mg.Kg^−1^ FfB, or 0.65 mg.Kg^−1^ FfB), or saline (*n* = 3/subgroup) followed by the retention test **(C)** or the extinction retention test **(D)**. The values are expressed as the means (±SEM). ^*^P < 0.05, ^**^P < 0.01 and ^***^P < 0.0001. ^###^*P* < 0.0001 according to ANOVA followed by *post-hoc* Bonferroni tests, when necessary.

The timelines illustrating the time points of drug administration and of brain removal are shown in Figures 2A, 3A, 4A and 5A. The effects of treatment with FfB and with agonists and antagonists specific to 5-HT_1A_Rs, GluN2B-NMDARs, and GABA_A_Rs or antagonists before FfB on the acquisition and extinction of the suppression of the licking response were assessed according to the mean SR for each tone, measured across 10 trials Figures 2B, 3B, 4B and 5B. Every figure shows the mean SRs for the CS on the first trial and each three-trial block from the retention test, extinction training, and extinction retention test sessions. The first trial is presented independently because it represents the first presentation of the CS after conditioning, extinction training, or retrieval of extinction; thus, the results from this trial can characterize the level of fear of the animal in each situation. In addition, the results from the first trial can show (i) the duration of fear memory expression and (ii) the occurrence of spontaneous recovery. The means (±SEM) for each first trial and block of three trials are presented in Tables S2, S4, S6 and S8.

To investigate the molecular mechanisms involved in modulating the suppression of the licking response by FfB, the expression levels of *Grin2a, Grin2b, Gabra5, Htr1a*, and *Erk2* in the DH were assayed by qRT-PCR. The effects of FfB, agonists and antagonists specific to the glutamatergic, serotoninergic and GABAergic systems were evaluated 3 h after the retention test session (8^th^ day; Figures 2C, 3C, 4C and 5C) and the extinction retention test session (10^th^ day; Figures 2D, 3D, 4D and 5D). The mean ± SEM values for the RE of candidate genes (ddCt) are available in Tables S3, S5, S7, S9.

Further, we have made statistical comparison between control groups, which received the vehicle solutions (Saline and Tween®). To comparison of SR means during all trial of presentation of CS we used Paired *t*-test. No statistically significant difference was found between-session or intra-session (*P* = 0.1450). To comparisons of differential gene expression from samples of DH, we have used unpaired *T*-test. Comparisons between Saline and Tween® groups, after retention test (8^th^ day), were made to each gene evaluated. No significant difference was observed in the RE levels of *Htr1a* (*P* = 0.9737), *Grin2b* (*P* = 0.9691), *Gabra5* (*P* = 0.9592), *Grin2a (P* = 0.7358), or *Erk2* (*P* = 0.0962). Comparisons between Saline and Tween® groups, after extinction retention test (10^th^ day), were made to each gene evaluated, similarly to aforementioned, no significant difference between groups was observed in the RE levels of *Htr1a* (*P* = 0.5834), *Grin2b* (*P* = 0.9208), *Gabra5* (*P* = 0.9982), *Grin2a* (*P* = 0.9628), or *Erk2* (*P* = 0.1469).

#### Effects of FfB on the acquisition and extinction of suppression of the licking response

The effects of FfB on the acquisition and extinction of suppression of the licking response are shown in Figure 2B and Table S2. A Two-way ANOVA revealed a significant group × trial interaction [*F*_(66, 756)_ = 2.785, *P* < 0.0001], a main effect of group [*F*_(66, 756)_ = 24.56, *P* < 0.0001] and a main effect of trial [*F*_(11, 756)_ = 17.27, *P* < 0.0001].

Comparisons of the results for the first trial in the retention test sessions between groups revealed elevated SR in the subgroups treated with FfB, CS-US or Tween and reduced SR in the subgroups treated with Sintocalmy® or CS (*P* < 0.0001; left panel of Figure 2B). The analysis of SR in the first three-trial block (2^nd^–4^th^ trials) showed a significant decrease in mean SR relative to the first trial in the Tween®, CS-US, and FfB subgroups (*P* < 0.0001); these results indicated the acquisition of extinction of fear memory within the session. An ANOVA comparing the three-trial blocks revealed no differences within sessions (*P* > 0.05). This finding indicated a reliable decrease in suppression and a reduction of fear after each session.

The middle panel of Figure 2B depicts the data from the extinction training session conducted 24 h after the retention test. Treatment with FfB at all doses promoted spontaneous recovery, as demonstrated by the results for the first trial in each subgroup, compared to treatment with Tween® or Sintocalmy® or to CS or CS-US alone. However, in subsequent trials, rats treated with FfB acquired fear extinction within the session (*P* < 0.0001). The Tween® and CS-US subgroups exhibited a similar SR mean across successive exposures to the CS during the extinction training session, as observed in the retention test. The Sintocalmy® and CS subgroups showed mean SRs during all CS presentations that were similar to those measured in the retention test (*P* > 0.05). This result indicated no conditioned fear and, as a consequence, no acquisition of fear extinction.

The right panel of Figure 2B shows the mean SRs in the extinction retention session conducted 24 h after extinction training. The subgroups treated with FfB exhibited spontaneous recovery in the first trial, similar to the behavior observed during the extinction training session. However, all subgroups exhibited similar behavior by the end of the session. Notably, for the first trials, the mean SRs for each subgroup treated with FfB were significantly different from those for the Tween® and CS subgroups (*P* < 0.0001). Comparisons between the first trial and the first three-trial block (2–4) showed reduced suppression of the licking response in the Tween®, CS-US and all FfB subgroups (*P* < 0.0001). An ANOVA comparing the results for the three-trial blocks within the session demonstrated no significant differences in the mean SR between the subgroups (*P* > 0.05). In addition, no significant differences in the mean SR were observed in the CS subgroup across all sessions (*P* > 0.05).

In summary, our data show for the first time that FfB does not impair the conditioned fear. However, rats treated with FfB showed spontaneous recovery of fear conditioning, as observed in the extinction training and extinction retention test sessions, although FfB did not prevent the acquisition within-session extinction. Furthermore, acute treatment with Sintocalmy®, a standardized extract containing 7% of the total flavonoids expressed in vitexin, impaired the conditioned fear and, consequently, resulted in no acquisition of the extinction of fear conditioning.

#### FfB treatment modulates the spontaneous recovery of fear memory via *Htr1a* and *Erk2* expression within the DH

FfB treatment at three different doses resulted in the overexpression of *Htr1a* [*F*_(7, 16)_ = 173.0, *P* < 0.0001], *Grin2a* [*F*_(7, 16)_ = 165.2, *P* < 0.0001], *Gabra5* [*F*_(7, 16)_ = 40.82, *P* < 0.0001], and *Erk2* [*F*_(7, 16)_ = 155.5, *P* < 0.0001] in the DH after the retention test session (8^th^ day) compared with the control treatments (Tween®, Sintocalmy®, CS-US, CS, and naïve; Figure 2C). The *Htr1a, Grin2b, Grin2a, Gabra5*, and *Erk2* expression levels were increased in the control subgroups (Tween® and CS-US subgroups) compared with the CS and naïve subgroups (*P* < 0.0001). No difference was observed in *Grin2b* [*F*_(7, 16)_ = 13.97] expression after treatment with FfB compared to the treatment with FfB and CS-US groups (*P* > 0.05; Figure 2C and Table S3), and *Grin2a* expression decreased after treatment with Sintocalmy®(*P* < 0.0001).

In the extinction retention test, *Htr1a* [*F*_(7, 16)_ = 96.39] and *Erk2* [*F*_(7, 16)_ = 388.9; *P* < 0.0001] expression was significantly increased after FfB treatment in the DH compared with all other treatments (*P* < 0.0001; Figure 2D and Table S3). No significant difference in the RE levels of *Grin2b* [*F*_(7, 16)_ = 12.20], *Gabra5* [*F*_(7, 16)_ = 16.11], or *Grin2a* [*F*_(7, 16)_ = 181.6] was observed in the FfB-treated subgroups compared to the CS-US or Tween® subgroups (*P* > 0.05), although these expression levels were increased compared to the CS and naïve subgroups (*P* < 0.05). Furthermore, *Grin2a* expression was reduced following the extinction retention test due to treatment with Sintocalmy® compared with all other treatments (Tween® and CS-US treatments; *P* < 0.0001).

In summary, the acquisition and extinction of the suppression of the licking response modulated *Htr1a, Grin2b, Grin2a, Gabra5*, and *Erk2* expression, and FfB treatment altered *Htr1a, Grin2a, Gabra5*, and *Erk2* expression after the retention test. Furthermore, the spontaneous recovery of fear memory appears to correlate with the overexpression of *Htr1a* and *Grin2a* in the DH.

#### Effects of FfB on fear memory after blocking 5-HT_1A_Rs

Figure 3B illustrates the specific effects of blocking 5-HT_1A_Rs before FfB treatment, which was administered before conditioning, on the results for the retention test, extinction training, and the extinction retention test. A Two-way ANOVA revealed a significant trial × group interaction [*F*_(55, 648)_ = 1.365, *P* = 0.0453], a main effect of group [*F*_(5, 648)_ = 2.792, *P* = 0.0166] and a main effect of trial [*F*_(11, 648)_ = 9.116, *P* < 0.0001]. Similar mean SRs were observed between the Tween and saline groups across sessions (*P* > 0.05). Therefore, saline was used to compare the effects of antagonists and agonists together with FfB.

The left panel of Figure 3B shows the mean SRs in the CS, negative control (saline), positive control (0.30 mg.Kg^−1^ (S)-WAY100135 and 10.0 mg.Kg^−1^ buspirone), and treated subgroups [0.30 mg.Kg^−1^ (S)-WAY+FfB (0.15 mg.Kg^−1^; 0.30 mg.Kg^−1^; or 0.65 mg.Kg^−1^FfB)] for the retention test session. The analysis of mean SR for the first trial showed that treatment with (S)-WAY100135 or (S)-WAY100135 before FfB resulted in reduced suppression of the licking response compared with saline (*P* < 0.0001) and buspirone treatment (*P* < 0.0001). Analysis of the SR for the first three-trial block (2^nd^–4^th^ trials) showed significant differences in the mean SRs compared to the first trial in the saline and buspirone subgroups (*P* < 0.0001). This result demonstrates acquisition within-session extinction to these subgroups. Alternatively no such differences were observed in the (S)-WAY100135 or (S)-WAY+FfB subgroups (*P* > 0.05). An ANOVA comparing the first three-trial block (2–4) with the subsequent three-trial blocks (5–7 and 8–10) demonstrated no significant differences in mean SR on the extinction retention session between the subgroups (*P* > 0.05; see Table S4).

The data from the extinction training tests are shown in the middle panel of Figure 3B. Comparisons between groups showed that the groups treated with (S)-WAY+FfB, at all doses, did not demonstrate a difference in the mean SR (*P* > 0.05). Analysis of the mean SR during the first three-trial block (2–4) showed that rats treated with (S)-WAY+FfB, saline, buspirone or (S)-WAY100135 exhibited a similar mean SR to that in the first trial (*P* > 0.05). Moreover, similar mean SRs were observed with in all groups for the subsequent three-trial blocks (5–7 and 8–10; *P* > 0.05; see also Table S4).

The data from the extinction retention tests are shown in the right panel of Figure 3B. The analysis of the SR showed that the subgroups treated with (S)-WAY100135+FfB did not exhibit spontaneous recovery. Furthermore, the analysis of the mean SR showed no significant difference between the first three-trial block (2^nd^–4^th^ trial) and the first trial among the (S)-WAY100135+FfB subgroups at all doses (*P* < 0.0001). Therefore, no significant differences in mean SRs were found among the subgroups between the first three-trial block (2–4) and the subsequent three-trial blocks (5–7 and 8–10; *P* > 0.05; see Table S4).

A Two-way ANOVA comparison between groups treated with FfB vs. (S)-WAY100135+FfB revealed a significant groups × trial interaction [*F*_(55, 495)_ = 2.018, *P* < 0.0001] and main effects of trial [*F*_(11, 99)_ = 21.25, *P* < 0.0001] and groups [*F*_(5, 45)_ = 21.41, *P* < 0.0001]. Treatment with (S)-WAY100135+ 030 mg.Kg^−1^ FfB and (S)-WAY100135+ 065 mg.Kg^−1^ FfB, resulted in reduced of licking response compared with FfB group in the first trial during extinction training. No significant difference was observed among subsequent three-trial blocks. Furthermore, the analysis of the mean SR showed significant difference between (S)-WAY100135+0.65 mg.Kg^−1^ FfB and 0.65 mg.Kg^−1^ FfB to the first trial during extinction retention test (*P* < 0.0001). No significant difference was observed among subsequent three-trial blocks.

In summary, our data demonstrate that (S)-WAY+FfB, at all doses, reduces the suppression of the licking response compared with the control treatment, as demonstrated by the results from the retention test. These data suggest for the first time that the spontaneous recovery observed in the FfB subgroups is modulated by 5-HT_1A_Rs.

#### (S)-WAY100135 treatment prevents the overexpression of *Htr1a* and *Erk2* within the DH caused by FfB

We used treatment with (S)-WAY100135 prior to FfB administration to assess the role of the 5-HT_1A_R in the acquisition and extinction of fear memory. In addition, the roles of NMDARs, GABA_A_R_S,_and ERK2 were evaluated.

Figure 3C shows the levels of *Htr1a, Gabra5, Grin2a, Grin2b*, and *Erk2* expression in the DH after the retention test session (8^th^ day). Consistent with the results of *Htr1a* and *Erk2* expression after FfB administration, treatment with (S)-WAY100135+FfB, at all doses, resulted in the downregulation of *Htr1a* expression [*F*_(5, 12)_ = 449.9, *P* < 0.0001]. Treatment with (S)-WAY100135+FfB resulted in the downregulation of *Htr1a* expression when compared with saline and buspirone treatment [*F*_(5, 12)_ = 40.05, *P* < 0.0001]. Although *Erk2* expression was similar across all groups [*F*_(5, 12)_ = 3.071, *P* = 0.0516]. A ANOVA comparison between the groups treated with (S)-WAY100135+FfB vs. FfB revealed that the overexpression of *Erk2* observed after FfB treatment was reversed by (S)-WAY100135 pretreatment, at all doses [*F*_(5, 12)_ = 57.79, *P* < 0.0001]. Furthermore, (S)-WAY100135+FfB induced the downregulation of *Grin2a* [*F*_(5, 12)_ = 124.8] and *Grin2b* [*F*_(5, 12)_ = 8.794;*P* = 0.001] expression compared with saline and buspirone (*P* < 0.0001). Moreover, (S)-WAY100135 treatment decreased the expression of *Grin2a*, but not *Grin2b*, and buspirone treatment reduced *Grin2b* expression compared with saline treatment (*P* < 0.0001). No significant changes in *Gabra5* expression were observed [*F*_(5, 12)_ = 2.505, *P* = 0.0894]. These statistics are shown in Table S5.

Figure 3D shows *Htr1a, Gabra5, Grin2a, Grin2b*, and *Erk2* expression in the DH after the extinction retention test (10^th^ day). These data show that treatment with (S)-WAY100135 before FfB administration, at three different doses, resulted in the overexpression of *Grin2a* [*F*_(5, 12)_ = 278.4, *P* < 0.0001] compared with the control treatments [saline, buspirone, and (S)-WAY100135] and in the downregulation of *Gabra5* compared to treatment with 0.15 mg.Kg^−1^ FfB [*F*_(5, 12)_ = 4.338, *P* = 0.0174]. Additionally, treatment with (S)-WAY100135+FfB resulted in the downregulation of *Htr1a* [*F*_(5, 12)_ = 31.18, *P* < 0.0001], *Erk2* [*F*_(5, 12)_ = 119.9, *P* < 0.0001] and *Gabra5* expression [*F*_(5, 12)_ = 20.48, *P* < 0.0001] and overexpression of *Grin2a* in the DH [*F*_(5, 12)_ = 82.00, *P* < 0.0001]. Furthermore, treatment with buspirone resulted in the upregulation of *Htr1a* expression [*F*_(5, 12)_ = 72.92; *P* < 0.001] compared with all other treatments. Nevertheless, no significant differences were observed in the RE of *Grin2b* [*F*_(5, 12)_ = 3.039, *P* = 0.0010] or *Erk2* [*F*_(5, 12)_ = 2.94, *P* = 0.0580]. Treatment with (S)-WAY100135 prior to FfB administration, at all three doses, prevented the upregulation of *Htr1a* expression observed after FfB treatment (Figure 2D; see also Table S5).

In summary, our data show that treatment with (S)-WAY100135 prior to FfB administration decreases *Htr1a, Grin2b*, and *Grin2a* expression in the DH after the retention test and prevents the increase in *Htr1a* and *Erk2* expression after the extinction retention test in relation to observed after treatment with FfB alone. Conversely, *Grin2a* expression in the DH was increased after (S)-WAY100135+FfB treatment after the extinction retention test compared with FfB treatment.

#### Effects of FfB on fear memory after blocking GluN2B-NMDARs

Figure 4B shows the effects of specifically blocking GluN2B-NMDARs with Ro25-6981 before FfB treatment, which was administered before conditioning, on the results from the retention test, extinction training and the extinction retention test. A Two-way ANOVA revealed a significant trial × group interaction [*F*_(55, 648)_ = 2.170, *P* < 0.0001], a main effect of group [*F*_(5, 648)_ = 3.356, *P* < 0.0001] and a main effect of trial [*F*_(11, 648)_ = 11.57, *P* < 0.0001].

The left panel of Figure 4B shows the mean SRs for the retention test session. Comparisons of the mean SR on the first trial revealed a difference between the Ro25-6981-treated subgroup and all other subgroups (*P* > 0.0001); this result indicated that blockade of GluN2B impaired the acquisition of fear memory. However, treatment with Ro25-6981 before FfB administration did not affect fear memory. FfB treatment reversed the learning impairment observed in the subgroup treated with Ro25-6981 alone. Analysis of the SR for the first three-trial block (2^nd^–4^th^ trials) showed significant differences in mean SR for the saline, NMDA, Ro25-6981, andRo25-6981+FfB groups (0.15 mg.Kg^−1^;0.30 mg.Kg^−1^; or 0.65 mg.Kg^−1^FfB) compared with the first trial (*P* < 0.0001). Furthermore, an ANOVA comparing the subsequent three-trial blocks (5–7 and 8–10) with the first three-trial block of the test revealed no differences (*P* > 0.05). The Ro25-6981 subgroup showed similar SR values across all trials of CS presentation (statistics shown in Table S6).

However, comparisons of the first trial of the extinction training test between the subgroups showed that the subgroups treated with Ro25-6981+FfB, at all doses, did not demonstrate differences in mean SR compared with the saline and NMDA subgroups (*P* > 0.05) or the Ro25-6981 alone subgroup, which showed no acquisition of conditioned fear (middle panel of Figure 4B). Analysis of the SR for the first three-trial block (2^nd^–4^th^ trials) compared with the first trial showed no significant differences in the mean SR for the subgroups treated with saline, NMDA, Ro25-6981, or Ro25-6981+FfB (*P* > 0.05). Nevertheless, no significant difference in SR was found within the groups for the first three-trial block (2–4) compared with the subsequent three-trial blocks (5–7 and 8–10; *P* > 0.05; see also Table S6).

The subgroups treated with Ro25-6981+FfB, at all doses, exhibited higher mean SRs for the first trial than the saline subgroup on the extinction retention test (*P* > 0.05; right panel of Figure 4B) and remained similar throughout the trials of the extinction training test. Comparisons between the first trial and the first three-trial block (2–4) showed reduced suppression of the licking response for all groups treated with Ro25-6981+FfB (*P* < 0.05). An ANOVA comparing the three-trial blocks demonstrated no significant differences in the mean SRs throughout the extinction retention session (*P* > 0.05; Table S6).

A Two-way ANOVA comparison between groups treated with FfB vs. Ro 25-6981+FfB revealed a significant groups × trial interaction [*F*_(55, 495)_ = 2.094, *P* < 0.0001] and main effects of trial [*F*_(11, 99)_ = 31.20, *P* < 0.0001] and groups [*F*_(5, 45)_ = 2.873, *P* = 0.0247). Analysis of the mean SR showed significant difference between Ro 25-6981+0.65 mg.Kg^−1^ FfB vs. 0.65 mg.Kg^−1^ FfB to the first trial during extinction training (*P* < 0.0001). No significant difference was observed among subsequent three-trial blocks (*P* > 0.05). Furthermore, similar means SR were found to groups treated with Ro 25-6981+FfB to the first trial during extinction retention test. No significant difference was observed among subsequent three-trial blocks (*P* > 0.05).

In summary, our data show for the first time that treatment with Ro25-6981, an antagonist of the GluN2B-NMDAR, impairs the acquisition of suppression of the licking response. Conversely, treatment with after FfB after Ro25-6981 administration, at all doses, reverses the learning impairment associated with the GluN2B-NMDAR antagonist. In this sense, GluN2B is involved in the acquisition of suppression of the licking response, but the disruptive effects of Ro25-6981 appear to be offset by treatment with FfB. Additionally, we showed that the spontaneous recovery observed in the FfB subgroups may be modulated by GluN2B because rats treated with Ro25-6981 before FfB administration seems to decrease the spontaneous recovery observed during the extinction training sessions compared with the rats treated with FfB alone (see Figure 2B).

#### Ro25-6981 treatment does not prevent the overexpression of *Grin2a* and *Erk2* caused by FfB, although it reduces *Htr1a* expression

We used treatment with Ro25-6981 prior to FfB to evaluate the roles of NMDARs, 5-HT_1A_R_*S*_, GABA_A_R_S_, and ERK2. Figure 4C shows the *Htr1a, Grin2a, Grin2b, Gabra5*, and *Erk2* expression levels in the DH after the retention test (8^th^ day). Treatment with Ro25-6981 before FfB treatment, at all three doses, resulted in the overexpression of *Grin2a* [*F*_(5, 12)_ = 107.1, *P* < 0.0001] and *Erk2* [*F*_(5, 12)_ = 90.89, *P* < 0.0001] and the decreased expression of *Htr1a* [*F*_(5, 12)_ = 32.67, *P* < 0.0001] and *Gabra5* [*F*_(5, 12)_ = 12.44, *P* = 0.0002] compared with the control treatment. No change in *Grin2b* was observed after Ro25-6981 or Ro25-6981+FfB treatment [*F*_(5, 12)_ = 20.18]. A ANOVA analysis revealed that treatment with Ro25-6981 before FfB, resulted in the overexpression of *Grin2a* [*F*_(5, 12)_ = 134.8, *P* < 0.0001] and *Erk2* [*F*_(5, 12)_ = 47.98, *P* < 0.0001] and the decreased expression of *Htr1a* [*F*_(5, 12)_ = 361.7, *P* < 0.0001] and *Gabra5* [*F*_(5, 12)_ = 32.57, *P* < 0.0001] in relation to groups treated with FfB alone. Additionally, NMDA treatment resulted in the overexpression of *Grin2b* compared with all other treatments (*P* < 0.0001) and in the overexpression of *Erk2* compared with saline or Ro25-6981 treatment (*P* < 0.001; see Table S7).

Figure 4D shows that treatment with Ro25-6981+FfB increased the *Grin2a* [*F*_(5, 12)_ = 14.47, *P* = 0.0001], *Erk2* [*F*_(5, 12)_ = 44.78, *P* < 0.0001], *Htr1a* [*F*_(5, 12)_ = 158.6, *P* < 0.0001], and *Grin2b* [*F*_(5, 12)_ = 5.37, *P* = 0.008] expression levels in the DH after the extinction retention test (10^th^ day) compared with saline treatment. No significant differences in *Gabra5* expression were observed between the subgroups treated with Ro25-6981+FfB and the saline subgroup [*F*_(5, 12)_ = 1.169, *P* = 0.3790]. Furthermore, comparison among groups treated with Ro25-6981 before FfB treatment resulted in the overexpression of *Grin2a* [*F*_(5, 12)_ = 32.68, *P* < 0.0001] and *Erk2* [*F*_(5, 12)_ = 34.58, *P* < 0.0001] and *Htr1a* [*F*_(5, 12)_ = 299.0, *P* < 0.0001]. Further, significant difference tog Grin2b was seeing to groups treated with Ro25-6981 before 0.65 mg.Kg^−1^ FfB in relation to 0.65 mg.Kg^−1^ FfB group [*F*_(5, 12)_ = 4.727, *P* = 0.0128].

In summary, treatment with Ro25-6981 reduced the expression of *Gabra5, Erk2*, and *Ht1ra* in the DH after the retention test, although treatment with FfB reduced the effects of Ro25-6981 on *Gabra5* and *Htr1a* expression and increased *Grin2a* and *Erk2* expression. Conversely, treatment with Ro25-6981+FfB increased *Htr1a* expression after the extinction retention test. Furthermore, treatment with FfB after Ro25-6981 administration increased the *Htr1a, Grin2b, Grin2a*, and *Erk2* expression levels in the DH after the extinction retention test (see Table S7).

#### Effects of FfB on fear memory after blocking GABA_A_Rs

The effects of specifically blocking GABA_A_Rs prior to FfB treatment before conditioning on the results of the retention test, extinction training, and the extinction retention test are shown in Figure 5B. A Two-way ANOVA revealed a significant trial × group interaction [*F*_(55, 648)_ = 2.695, *P* < 0.0001], a main effect of group [*F*_(5, 648)_ = 6.416, *P* < 0.0001] and a main effect of trial [*F*_(11, 648)_ = 11.77, *P* < 0.0001].

Analysis of the mean SRs for the first trial of the retention test showed that treatment with picrotoxin, an antagonist of GABA_A_Rs, or picrotoxin+FfB did not prevent the acquisition of conditioned fear. These subgroups showed a similar mean SR to the saline subgroup (*P* > 0.05). Conversely, animals treated with diazepam exhibited reduced suppression of the licking response compared with the animals treated with saline, picrotoxin or picrotoxin+FfB (*P* < 0.001).

Analysis of the SR for the first three-trial block (2^nd^–4^th^ trials) compared with the first trial showed significant differences in the mean SR for the subgroups treated with saline, picrotoxin, or picrotoxin+FfB (0.15 mg.Kg^−1^, 0.30 mg.Kg^−1^, or 0.65 mg.Kg^−1^FfB; *P* < 0.0001). An ANOVA comparing the first three-trial block (2–4) of extinction with the other three-trial blocks (5–7 and 8–10) demonstrated no significant differences in the mean SR between the subgroups (*P* > 0.05; see Table S8).

The data from the extinction training session are shown in the middle panel of Figure 5B. Comparisons between the groups showed that the subgroups treated with picrotoxin+FfB, at all doses, demonstrated differences in mean SRs in the first trial compared to the saline, picrotoxin and diazepam subgroups (*P* < 0.0001). In addition, rats treated with picrotoxin+FfB showed spontaneous recovery similar to that observed in rats treated with FfB alone (see Figure 2B). Furthermore, no differences in SR on the first trial were observed between the saline and picrotoxin subgroups (*P* > 0.05). Analysis of the mean SR during the first three-trial block (2–4) showed that rats treated with picrotoxin+FfB at all doses demonstrated reduced suppression of the licking response compared with the mean SR for the first trial (*P* < 0.0001). The saline and picrotoxin subgroups exhibited a similar mean SR across successive exposures to the CS during extinction training. Similar mean SRs were observed for all groups across the subsequent three-trial blocks (5–7 and 8–10; *P* > 0.05; see Table S8).

Similar to the previous sessions, on the extinction retention test, rats treated with picrotoxin+FfB, at all doses, showed spontaneous recovery on the first trial, as demonstrated by the higher SR means in the picrotoxin+FfB subgroups (*P* > 0.001; Figure 5B, right panel). A reduced mean SR was observed in the picrotoxin+FfB subgroups on the first three-trial block compared to the first trial (*P* < 0.0001). No significant difference in the SR was found within the groups for the first three-trial block (2–4) compared with the subsequent three-trial blocks (5–7 and 8–10; *P* > 0.05; see also Table S8).

A Two-way ANOVA comparison between groups treated with FfB vs. Picrotoxin+FfB revealed a significant groups × trial interaction [*F*_(55, 495)_ = 1.091, *P* = 0.3116] and main effects of trial [*F*_(11, 99)_ = 45.11, *P* < 0.0001] and groups [*F*_(5, 45)_ = 5.375, *P* = 0.0006]. Analysis of the mean SR showed no significant difference between groups treated with FfB vs. Picrotoxin+FfB, at all doses, to the first trial during retention test (*P* > 0.05), extinction training (*P* > 0.05) and extinction test sessions (*P* > 0.05). Significantly difference were observed among groups treated with FfB, at a dose 0.15 mg.Kg^−1^ and 0.65 mg.Kg^−1^FfB (*P* < 0.05). Furthermore, analysis of SR means for the first trial showed significant difference among rats treated with different doses of FfB groups during extinction training and extinction retention test (*P* < 0.001). No significant difference was observed among subsequent three-trial blocks (*P* > 0.05).

In summary, our data demonstrated that each group treated with picrotoxin prior to FfB administration acquired fear memory. Conversely, diazepam treatment impaired the acquisition of fear memory. Furthermore, we showed that treatment with picrotoxin+FfB resulted in spontaneous recovery in the first trial of extinction training and the extinction retention test, although the suppression gradually decreased over the trials. Therefore, rats treated with FfB showed within-session extinction of fear memory. These data suggest that spontaneous recovery is not modulated by GABA_A_Rs.

#### Picrotoxin prevents the overexpression of *Gabra5* and *Grin2a* caused by FfB

Treatment with picrotoxin prior to FfB treatment, at a dose of 0.15 or 0.65 mg.Kg^−1^ FfB, did not prevent the increase in the expression of *Htr1a* [*F*_(5, 12)_ = 28.02, *P* < 0.0001] or *Erk2* [*F*_(5, 12)_ = 84.48, *P* < 0.0001] in the DH after the retention test compared with saline, picrotoxin, or diazepam treatment (Figure 5C), as observed in the subgroups treated with FfB alone (Figure 2C). A ANOVA analysis revealed that treatment with picrotoxin before FfB, resulted in the downexpression of *Htr1a* [*F*_(5, 12)_ = 128.7 *P* < 0.0001], *Grin2b* [*F*_(5, 12)_ = 15.22, *P* < 0.0001] and *Erk2* [*F*_(5, 12)_ = 22.81, *P* < 0.0001], at all doses, and *Gabra5* [*F*_(5, 12)_ = 28.02, *P* < 0.0001] and *Grin2a* [*F*_(5, 12)_ = 8515, *P* < 0.00002], at a higher doses in relation to groups treated with FfB alone. Additionally, picrotoxin+FfB treatment did reduce *Grin2b* expression [*F*_(5, 12)_ = 14.42, *P* < 0.0001], but no change was observed in the expression of *Grin2a* [*F*_(5, 12)_ = 12.24, *P* = 0.0001] or *Gabra5* [*F*_(5, 12)_ = 7.580, *P* = 0.0020]. Furthermore, picrotoxin increased *Gabra5*, and *Erk2* expression in the DH (*P* < 0.0001). Conversely, diazepam treatment decreased *Htr1a, Grin2a, Erk2*, and *Grin2b* expression in the DH (see Table S9).

The data shown in Figure 5D demonstrate the upregulation of *Erk2* [*F*_(5, 12)_ = 90.76, *P* < 0.0001] and *Grin2a* [*F*_(5, 12)_ = 67.51] expression in the DH after the extinction retention session for the subgroups treated with picrotoxin+*F*fB compared to those treated with saline, picrotoxin or diazepam (*P* > 0.0001). Upregulated *Htr1a* [*F*_(5, 12)_ = 23.98, *P* < 0.0001] and *Erk2* [*F*_(5, 12)_ = 26.24, *P* < 0.0001] expression was observed in the picrotoxin+FfB in relation to FfB group. Similarly, upregulated *Htr1a* [*F*_(5, 12)_ = 10.75] expression was observed in the picrotoxin+FfB compared with the saline and diazepam subgroups (*P* < 0.0001). Moreover, we showed that picrotoxin resulted in the overexpression of *Htr1a, Grin2b* [*F*_(5, 12)_ = 31.61, *P* < 0.0001], and *Gabra5* [*F*_(5, 12)_ = 12.13, *P* = 0.0020] compared with saline. Downregulated *Grin2b* [*F*_(5, 12)_ = 22.08, *P* < 0.0001], *Grin2a* [*F*_(5, 12)_ = 22.04, *P* < 0.0001] and *Gabra5* [*F*_(5, 12)_ = 14.46, *P* < 0.0001] expression was observed in the picrotoxin+FfB in relation to FfB group. Furthermore, diazepam increased *Htr1a* expression and decreased *Grin2a* and *Gabra5* expression (*P* < 0.0001; see Table S9).

In summary, treatment with picrotoxin before FfB administration, at all doses, increased *Htr1a* and *Erk2* expression in the DH after the acquisition and extinction of fear memory and reduced *Grin2b* expression and prevented the increase in *Grin2a* and *Gabra5* expression after the retention test. Furthermore, this treatment decreased *Grin2b* and *Gabra5* expression after the extinction retention test.

## Discussion

The major findings of our study are as follows. (i) Rats treated with FfB acquired suppression of the licking response, and FfB upregulated the expression of *Htr1a, Grin2a, Gabra5*, and *Erk2* in the DH after the acquisition of conditioned fear, compared to rats exposed to the CS alone, naïve rats and Sintocalmy®-treated rats. (ii) Rats treated with FfB, at all doses, showed spontaneous recovery when subjected to the extinction training and extinction retention test sessions; these observations were correlated with *Htr1a* and *Erk2* overexpression in the DH. (iii) These findings were confirmed by data from treatment with (S)-WAY100135, which reduced the lick SR and inhibit spontaneous recovery. Further, data from DH samples obtained from rats treated with (S)-WAY100135 prior to FfB resulted in the downregulation of *Htr1a* expression and no modulation of *Erk2* expression after the retention test and the extinction retention test. (iv) Our data are in line with previous findings concerning the requirement of GluN2B for fear memory formation (Sotres-Bayon et al., 2007, 2009). In particular, we present evidence that treatment with Ro25-6981 disrupts the acquisition of suppression of the licking response. Nevertheless, treatment with FfB after Ro25-6981 reversed the dose-dependent deficit in the acquisition of fear memory caused by Ro25-6981, which was associated with upregulation of *Grin2*a and *Erk2* expression and downregulation of *Htr1a* and *Gabra5* expression in the DH after the retention test. The occurrence of spontaneous recovery to group treated with Ro25-6981 before FfB during extinction retention test seems to be associated with increase of *Grin2b, Grin2a*, and *Erk2* expression. (v) Treatment with picrotoxin prior to FfB administration no inhibits the spontaneous recovery of fear. This observation was correlated with overexpression of *Htr1a* and *Erk2* and no modulation of *Gabra5* expression in the DH. This result suggested that spontaneous fear recovery is not modulated by inactivation of GABA_A_Rs; however, the data concerning *Gabra5* expression in the DH indicated that FfB modulated the expression of the α5-subunit, which is particularly important for mediating the process of memory formation in the hippocampus (Bannerman et al., 2004; Rudolph and Möhler, 2006; Atack, 2011). Additionally, treatment with diazepam and Sintocalmy® disrupt the acquisition of fear memory, in which was associated with downregulation of *Grin2a* expression in the DH. Several pharmacological studies have indicated that the administration of diazepam before training impairs LTM, as evaluated in a behavioral model such as IA (Izquierdo and Ferreira, 1989), contextual fear conditioning (Harris and Westbrook, 1998), or conditioned suppression (Oliveira et al., 2009). Consistent with this evidence, our results show that acute treatment with 4.0 mg.Kg^−1^ diazepam impaired fear memory acquisition and highlight the role of GABA_A_R in this process. Together with previous data, our current data further support the concept that flavonoid fractions do not prevent fear memory extinction within a session (de Oliveira et al., 2014). In addition, these data suggest an important role of the DH in mediating the acquisition and extinction of conditioned suppression of the lick response.

The roles of the hippocampus in the acquisition, consolidation, and retrieval of fear memory (Kim and Fanselow, 1992; Cammarota et al., 2008) and in fear extinction have been extensively studied in different rodent paradigms (Izquierdo, 1997; Ji and Maren, 2008). Further, the involvement of a circuit including the hippocampus, the pre-frontal cortex and the amygdala in these processes has long been established (Vinogradova, 2001; Fanselow and Dong, 2010). However, the present data suggest an important role of the hippocampus in conditioned suppression, whereas hippocampal plasticity may represent another function of the hippocampus in addition to contextual fear memory modulation and executive and integrative functions (McNaughton and Gray, 2000; Anagnostaras et al., 2001; Vinogradova, 2001; Sanders et al., 2003). Further, many theories have attempted to explain both the neurochemical processes that occur during the acquisition and extinction of fear memory and in the mechanism by which new drug, which are designed to enhance the consolidation or facilitate the extinction of fear memories, might modulate these neurochemical systems (Ji and Maren, 2007; Dalton et al., 2008). However, much less is known about drugs that modulate the brain substrates of extinction, conditioned inhibition, and other inhibitory processes involved in the suppression of a motivated response or the basis of spontaneous recovery. In addition, very few studies have shown the effects of drug treatment prior to conditioning training on fear extinction or spontaneous recovery, i.e., the relationship between the strength of fear memory acquisition and spontaneous recovery. In contrast, the majority of the existing data show the effects of pre-extinction treatment on spontaneous recovery.

Our data suggest that the role of the hippocampus in the acquisition and extinction of lick suppression is dependent on the interaction between glutamatergic, serotoninergic and GABAergic neurotransmission via the activation or inactivation of specific NMDARs, GABA_A_Rs, and 5-HT_1A_Rs, as demonstrated by the results from pharmacological manipulation and differential gene expression of *Grin2a, Grin2b, Gabra5, Htr1a*, and *Erk2*. The reappearance of a conditioned response after acquisition and training for extinction of fear memory, as shown in our subgroups treated with FfB, has been previously described (Bouton, 1993; Rescorla, 2004; Leung and Westbrook, 2008; Quirk and Mueller, 2008). Specifically, it is thought that the persistence of a fear response after extinction training is associated with anxiety-related disorders (Davis et al., 2006). However, we showed that FfB enabled the acquisition of extinction within a session despite the occurrence of spontaneous recovery. Although these findings may seem paradoxical, our current findings raise the hypothesis that the original memory was somewhat enhanced, i.e., better preserved; therefore, the flavones from *Erythrina falcata* may be studied as a novel pharmacotherapy for the treatment of cognitive impairment. Furthermore, we believe that the reappearance of the original memory (spontaneous recovery) observed after FfB treatment is associated with the expression of *Htr1a, Erk2*, and *Grin2a* in the DH.

### 5HT_1A_Rs as a potential target for the effects of FfB on spontaneous recovery

Drugs that modulate the serotoninergic system are important for cognitive and emotional functions, and 5-HT_1A_Rs are involved in this process. The heteromeric 5-HT_1A_R is highly expressed in the hippocampus (Barnes and Sharp, 1999), where it modulates GABA- and glutamate-mediated activities (Jacobs and Azmitia, 1992; Barnes and Sharp, 1999; Meneses and Perez-Garcia, 2007). Activation of post-synaptic 5-HT_1A_Rs (heteroreceptors) in the hippocampus is a central component of conflict resolution and anti-anxiety effects. Alternatively, reduced 5-HT_1A_R expression results in a deficit in hippocampal-dependent memory (Bert et al., 2005, 2006; Altieri et al., 2013). However, the effect of the activation of 5-HT_1A_R on the modulation of *Erk*2 expression remains controversial and may depend on neuronal origin and maturation states. Treatment with a 5-HT_1A_R agonist increased ERK phosphorylation and activity in the hippocampal neuron-derived cell line HN2-5 and in hippocampal slices cultured from postnatal day-15 animals (Adayev et al., 1999). In addition to these effects, the activation of 5-HT_1A_Rs alters the dynamics of other neurotransmission systems.

The serotonergic regulation of NMDAR function in the DH was described in pyramidal neurons in the prefrontal cortex (Yuen et al., 2005). Additionally, the activation of 5-HT_1A_Rs resulted in disruption of the transport of GluN2B subunit-containing vesicles in dendrites, and this transport is regulated by the CaMKII and ERK signaling pathways (Yuen et al., 2005). However, further investigations of the adaptive changes in receptor functions and their specific localization are needed to elucidate the precise role of flavonoids.

Intra-hippocampal treatment with (S)-WAY100135 alone did not affect the punished response in rats (Przegalinski et al., 1995). Therefore, our data suggest that treatment with (S)-WAY100135 reduced lick suppression and that treatment with FfB was unable to reverse this effect. Moreover, the treatment with (S)-WAY100135 modulated *Grin2a* and *Grin2b* expression. In this sense, heteromeric 5-HT_1A_Rs in the DH appear to be related to the acquisition of conditioned fear in addition to anti-conflict functions because rats treated with (S)-WAY100135 before FfB administration did not show spontaneous recovery.

The reduced *Grin2a, Grin2b*, and *Htr1a* expression in the DH in groups treated with (S)-WAY100135 or (S)-WAY+FfB may underlie the reduced lick suppression and lack of spontaneous recovery. This result suggests an interaction between neurochemical systems. Therefore, the 5-HT_1A_R represents an additional potential target for the regulation of emotion and cognition in the DH.

### Activation of the GluN2B-NMDARs is required for acquisition of conditioned suppression and their inactivation before FfB treatments modulates the spontaneous recovery

Since the discovery of the involvement of NMDARs in long-term potentiation (LTP) at CA1 synapses in the hippocampus, it has become evident that NMDARs are critical for a variety of cognitive processes, such as the acquisition and extinction of fear conditioning (Morris et al., 1986; Bliss and Collingridge, 1993). GluN2A and GluN2B are the predominant subunits of NMDARs. Furthermore, both of these subunits are expressed in the adult brain, predominantly in forebrain regions such as the amygdala, the prefrontal cortex, and the hippocampus, which are involved in the signaling pathways required for aversive memory formation (Schenberg et al., 2006; Mathur et al., 2009; Sotres-Bayon et al., 2009; Morris, 2013).

The hippocampal functions of NMDARs, particularly the GluN2B and GluN2A subunits, in fear memory have been reported (Zhang et al., 2008; Brigman et al., 2010). Several works have suggested that the NMDAR subunit composition could be responsible for the induction of the two forms of plasticity: LTP and long-term depression (LTD) (Shipton and Paulsen, 2014). The contribution of each subunit to ERK2 activation appears to be related to the localization and population of these receptors as well as the behavioral paradigm evaluated (Traynelis et al., 2010). NMDARs either produce weak ERK2 activation or do not activate ERK2 (Gao et al., 2010). Myung et al. (2005) showed that the GluN2B-NMDAR is coupled to the inhibition, rather than the activation, of ERK1/2. Furthermore, differences between behavioral data and gene expression data may explain the different effects of the GluN2B-NMDAR on downstream pathways according to regional localization. Our data showed that Ro25-6981 downregulated the expression of *Erk2* in the DH, which resulted in the impairment of conditioned suppression. Alternatively, pharmacological activation of NMDARs increased *Grin2b* and *Erk2* expression but did not affect *Grin2a* expression in the DH after the acquisition of conditioned suppression. Furthermore, treatment with FfB after Ro25-6951 administration increased *Grin2a* and *Erk2* expression in the DH. Thus, *Erk2* activity is closely related to the acquisition of conditioned suppression, as well as extinction and spontaneous recovery.

The increase in *Erk2* expression, in response to the acquisition of fear memory or to NMDAR stimulation, has been consistently related to memory-dependent plasticity in the hippocampus (Atkins et al., 1998; Cammarota et al., 2000). The first evidence for the involvement of MAPK in LTP and fear memory originated from studies by English and Sweatt (1996) and Atkins et al. (1998), which showed that ERK2 is required for the formation of LTM in a fear conditioning paradigm in the hippocampus. The levels of ERK2 are elevated following the activation of NMDARs and during the influx of calcium (Impey et al., 1999) but are decreased by 5-HT_1A_-receptor activation or infusion of an agonist of the serotonergic 5-HT_1A_R in the hippocampus as Erk1/2 plays an important role in neuroprotection and synaptic activity.

In addition to hippocampal NMDARs and 5-HT_1A_Rs, GABA_A_Rs play an important role in synaptic plasticity and therefore contribute to the acquisition of fear memory. Accordingly, drugs that modulate GABAergic transmission have been shown to interfere with fear acquisition and extinction (Chhatwal et al., 2005; Delamater et al., 2009; Oliveira et al., 2009).

### Activation of GABA_A_Rs impairs acquisition of conditioned suppression. their inactivation, before FfB treatment, however, didn't prevent the spontaneous recovery

We observed that conditioned suppression was impaired in the subgroups treated with Sintocalmy® or diazepam, and this impairment appeared to be related to the downregulation of *Grin2a* expression in the DH. The pharmacological properties and behavioral actions of benzodiazepines, such as amnesic, sedative, and antianxiety effects, on GABA_A_Rs appear to be mediated by the α1 subunit, which is preferentially located in interneurons of forebrain areas (Collinson et al., 2002). However, evidence has demonstrated that the GABA_A_R α5 subunit is highest in the hippocampus compared with deep cortical layers and the amygdala (Rudolph and Möhler, 2014), where it mediates memory formation (Yee et al., 2004; Rudolph and Möhler, 2006; Atack, 2011) and is involved in learning and memory tasks (Harris and Westbrook, 1998; Collinson et al., 2002, 2006). Although *Gabra5* expression was not modulated in rats subjected to fear conditioning with or without FfB treatment or to the acquisition of conditioned suppression following FfB treatment, rats treated with picrotoxin displayed upregulation of *Gabra5* expression and showed acquisition of memory. In addition to the role of the α5 subunit in the acquisition of fear memory, its modulation in the DH after extinction of fear memory is supported by data from the subgroups treated with picrotoxin+FfB; these data suggest that the α5 subunit is not correlated with spontaneous recovery. Thus, our data reveal a central role of the α5 subunit of the GABA_A_R in the acquisition of conditioned emotional suppression, as evaluated by the lick response. The memory-enhancing effects of benzodiazepine site partial inverse agonists have been shown (Yee et al., 2004).

## Conclusion

The major fear memory/treatment-dependent changes observed in our study included the spontaneous recovery of fear memory, which may be related to the enhancement of consolidation of fear memory. No anti-anxiety effects were observed after treatment with FfB. Furthermore, for the first time, we showed that the spontaneous recovery of fear memory may be correlated with the combined activation of GluN2A-containing NMDARs, and 5-HT_1A_Rs in the DH, which, in turn, modulates ERK1/2 activity. Finally, the results from gene expression analysis in the DH and the results showing the modulatory effects of FfB treatment indicate that the DH appears to anatomically and functionally subserve other structures involved in the acquisition and extinction of fear memory formation, such as the amygdala and the prefrontal cortex. Together, our data provide important information concerning the molecular basis of fear-conditioned suppression and the role of the DH in these processes, and our results suggest that FfB may represent a potential therapeutic target for preventing or treating neurocognitive impairments.

### Conflict of interest statement

The authors declare that the research was conducted in the absence of any commercial or financial relationships that could be construed as a potential conflict of interest.

## Supplementary material

Figure S1 **HPLC-DAD-ESI/MS^**n**^ analysis of the flavonoidic fraction (FfB) from the roots of ***Erythrina falcata*** using a C18 Luna column (A,B)**. TIC was performed in negative mode, and MS^2^ spectra of deprotonated molecules **(A)** were obtained as follows: (1) vicenin-2 [M-H]^−^ at *m/z* 593, (2) vicenin-1 [M-H]^−^ at *m/z* 563, (3) vitexin [M-H]^−^ at *m/z* 431, (4) isovitexin [M-H]^−^ at *m/z* 431, (5) 6-C-glycoside diosmetin [M-H]^−^ at *m/z* 461, and (6) apigenin [M-H]^−^ at *m/z* 269. The chromatogram was recorded at 254 nm for the UV spectra of compounds 1-6 **(A)**.
