# Supplementary figures and images for: Effects of a Flavonoid-Rich Fraction on the Acquisition and Extinction of Fear Memory: Pharmacological and Molecular Approaches
Source: Front Behav Neurosci. 2016 Jan 5;9:345. doi: 10.3389/fnbeh.2015.00345 (PMC4700274; doi:10.3389/fnbeh.2015.00345)

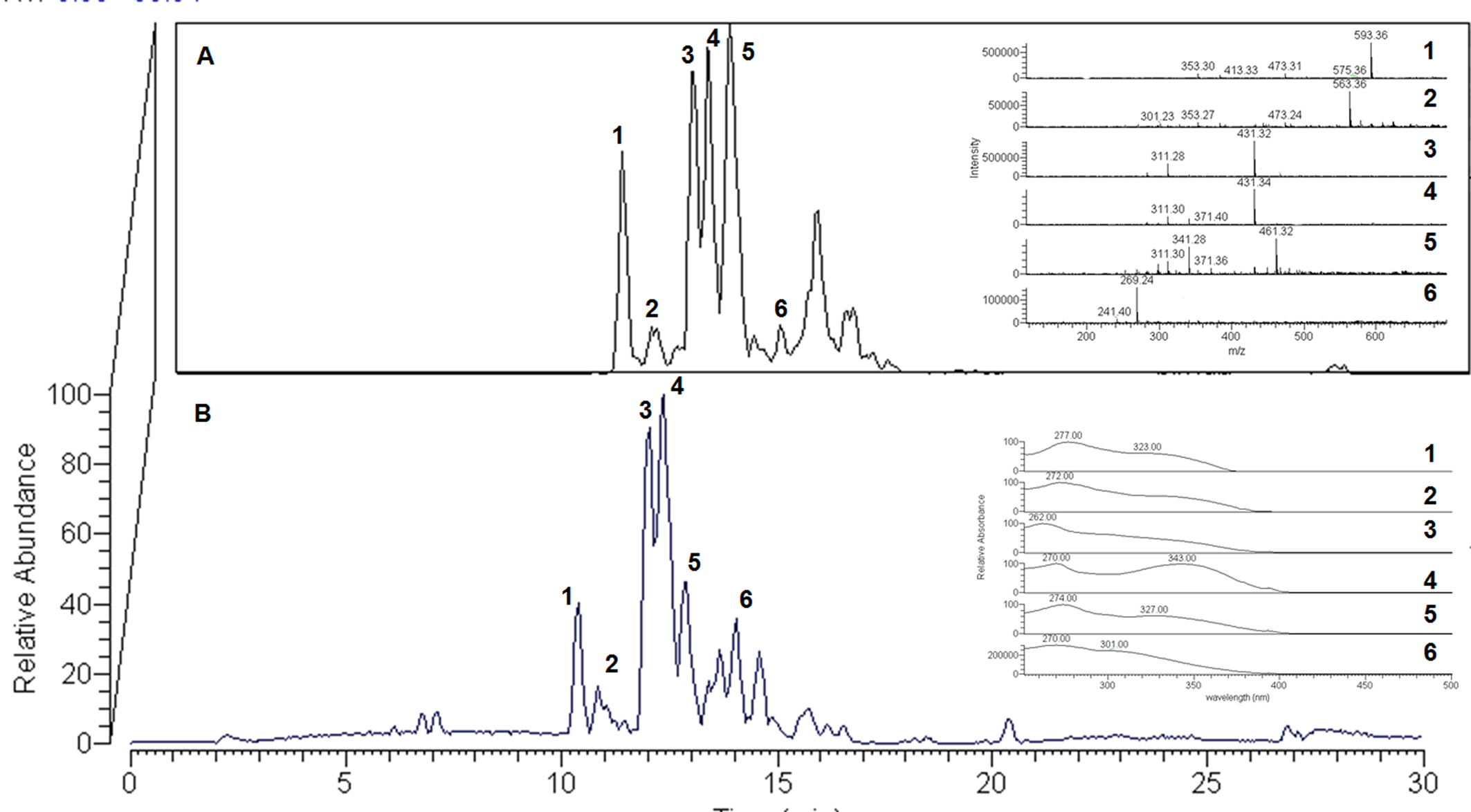

Supplement: Figure S1 — HPLC-DAD-ESI/MSn analysis of the flavonoidic fraction (FfB) from the roots of Erythrina falcata using a C18 Luna column (A,B). TIC was performed in negative mode, and MS2 spectra of deprotonated molecules (A) were obtained as follows: (1) vicenin-2 [M-H]− at m/z 593, (2) vicenin-1 [M-H]− at m/z 563, (3) vitexin [M-H]− at m/z 431, (4) isovitexin [M-H]− at m/z 431, (5) 6-C-glycoside diosmetin [M-H]− at m/z 461, and (6) apigenin [M-H]− at m/z 269. The chromatogram was recorded at 254 nm for the UV spectra of compounds 1-6 (A). [file Image1.TIF]
