# Supplementary material for: Effects of a Flavonoid-Rich Fraction on the Acquisition and Extinction of Fear Memory: Pharmacological and Molecular Approaches
Source: Front Behav Neurosci. 2016 Jan 5;9:345. doi: 10.3389/fnbeh.2015.00345 (PMC4700274; doi:10.3389/fnbeh.2015.00345)
Supplement: Supplementary file 2 [file Table1.DOCX]

**Table S1-**Primers used in this study.

| **Gene** | **Primer (5'-3')** | **PCR product size (pb)** |
| --- | --- | --- |
| **RPS8** | F- CGTGCTCTGAGATTGGATGT | 109 |
|  | R- CGGACAAGCTCGTTATTGG |  |
| ***Htr1a*** | F- CCGCACGCTTCCGAATCC | 109 |
|  | R- TGTCCGTTCAGGCTCTTCTTG |  |
| ***Grin2b*** | F-AAGCCTGGCATGGTCTTCTC | 112 |
|  | R-TGTTGTTCATGGTGGCAGTG |  |
| ***Grin2a*** | F- GGTGAATTTCTTCCCCAGCA | 80 |
|  | R-ACAATGCAGGCAGCTCAGAA |  |
| ***Mapk1/Erk2*** | F-TCGATTCCAGCCAGGATACA | 107 |
|  | R-AGACAGGACCAGGGTCAAGAA |  |
| ***Gabra5*** | F-TGTCTTCTCTGCCCTGATTG | 173 |
|  | R-TTGGAGGATGGGTCAACTTC |  |
