# Supplementary material for: Effects of a Flavonoid-Rich Fraction on the Acquisition and Extinction of Fear Memory: Pharmacological and Molecular Approaches
Source: Front Behav Neurosci. 2016 Jan 5;9:345. doi: 10.3389/fnbeh.2015.00345 (PMC4700274; doi:10.3389/fnbeh.2015.00345)
Supplement: Supplementary file 3 [file Table2.DOCX]

**Table S2-** Mean SR to the CS (tone) for the first trial and three-trial blocks in the retention test, extinction training and extinction retention test for the control groups (CS, learning, Tween^®^ and Sintocalmy^®^) and the groups treated with FfB (0.15 mg.Kg^-1^,0.30 mg.Kg^-1^or 0.65 mg.Kg^-1^).

| **GROUPS** | **TRIALS** | | | | | | | | | | | |
| --- | --- | --- | --- | --- | --- | --- | --- | --- | --- | --- | --- | --- |
|  | **Retention test (8^th^ day)** | | | | **Extinction training (9^th^ day)** | | | | **Extinction retention test (10^th^ day)** | | | |
|  | **1** | **2 - 4** | **5-7** | **8-10** | **1** | **2 - 4** | **5-7** | **8-10** | **1** | **2 – 4** | **5-7** | **8-10** |
| CS (a) | 0.51 ± 0.03 | 0.50 ± 0.10 | 0.54 ± 0.11 | 0.54 ± 0.15 | 0.48 ± 0.04 | 0.49 ± 0.03 | 0.50 ± 0.04 | 0.51 ± 0.02 | 0.49 ± 0.04 | 0.52 ± 0.02 | 0.46 ± 0.02 | 0.46 ± 0.04 |
| Learning (b) | 0.75± 0.17^a,d^ | 0.53 ± 0.02^###^ | 0.55 ± 0.02 | 0.55 ± 0.01 | 0.57± 0.05 | 0.55 ± 0.03 | 0.56 ± 0.03 | 0.55 ± 0.02 | 0.45 ± 0.02 | 0.49 ± 0.02 | 0.47 ± 0.01 | 0.45 ± 0.02 |
| Tween^®^ (c) | 0.72±0.05^a,d^ | 0.56 ± 0.02^###^ | 0.51 ± 0.02 | 0.59 ± 0.02 | 0.51± 0.03 | 0.54 ± 0.03 | 0.52 ± 0.03 | 0.58 ± 0.03 | 0.41 ± 0.05 | 0.44 ± 0.03 | 0.49 ± 0.02 | 0.49 ± 0.03 |
| 600 mg.Kg-^1^ Sintocalmy^®^ (d) | 0.48 ± 0.05 | 0.46 ± 0.03 | 0.47 ± 0.04 | 0.49 ± 0.02 | 0.48± 0.05 | 0.46 ± 0.02 | 0.48 ± 0.04 | 0.49 ± 0.01 | 0.43 ± 0.09 | 0.48 ± 0.03 | 0.48 ± 0.03 | 0.49 ± 0.02 |
| 0.15 mg.Kg^-1^FfB (e) | 0.71 ± 0.03^a,d^ | 0.59±0.03^###^ | 0.55 ± 0.02 | 0.59 ± 0.02 | 0.69±0.01^a,b,c,d^ | 0.57 ± 0.01^###^ | 0.56 ± 0.01 | 0.57 ± 0.02 | 0.58 ± 0.06^a,b,c,d^ | 0.51 ± 0.06^###^ | 0.53 ± 0.04 | 0.48 ± 0.03 |
| 0.30 mg.Kg^-1^FfB (f) | 0.74± 0.03^a,d^ | 0.57±0.02^###^ | 0.58 ± 0.01 | 0.62 ± 0.02 | 0.73±0.01^a,b,c,d^ | 0.54 ± 0.02^###^ | 0.57 ± 0.03 | 0.58 ± 0.02 | 0.66± 0.02^a,b,c,d^ | 0.55± 0.05^###^ | 0.55 ± 0.03 | 0.49 ± 0.03 |
| 0.65mg.Kg^-1^FfB (g) | 0.73± 0.03^a,d^ | 0.51±0.02^###^ | 0.55± 0.05 | 0.52± 0.01 | 0.82±0.01^a,b,c,d^ | 0.51 ± 0.08^###^ | 0.52 ± 0.05 | 0.56 ± 0.06 | 0.70 ± 0.02^a,b,c,d^ | 0.46 ± 0.04^###^ | 0.45 ± 0.05 | 0.42 ± 0.06 |

The results are presented as the means (±SEM). Inter-group and inter-trial comparisons

were computed and can be evaluated.

^a^*P*<0.0001 – Comparison of SR for the first trial for each group x CS group.

^b^*P*<0.0001 – Comparison of SR for the first trial for each group x learning group.

^c^*P*<0.0001 – Comparison of SR for the first trial for each group x Tween^®^ group.

^d^*P*<0.0001 – Comparison of SR for the first trial for each group x Sintocalmy^®^ group.

^###^ *P*<0.0001 – Comparison of SR for the first-trial x the first three-trial block (2^nd^-4^th^ trials) for each group.
