# Supplementary material for: Effects of a Flavonoid-Rich Fraction on the Acquisition and Extinction of Fear Memory: Pharmacological and Molecular Approaches
Source: Front Behav Neurosci. 2016 Jan 5;9:345. doi: 10.3389/fnbeh.2015.00345 (PMC4700274; doi:10.3389/fnbeh.2015.00345)
Supplement: Supplementary file 4 [file Table3.DOCX]

**Table S3–***Htr1a, Grin2b, Grin2a, Gabra5* and *Mapk1-Erk2* expression in the DH by qRT-PCR after the retention test and the extinction retention test for the control groups (CS, learning, Tween^®^ and Sintocalmy^®^) and the groups treated with FfB (0.15 mg.Kg^-1^, 0.30 mg.Kg^-1^or 0.65 mg.Kg^-1^).

| **GROUPS** | **Relative expression (ddCt)** | | | | | | | | | |
| --- | --- | --- | --- | --- | --- | --- | --- | --- | --- | --- |
|  | **Retention test (8^th^ day)** | | | | | **Extinction retention test (10^th^ day)** | | | | |
|  | ***Htr1a*** | ***Grin2b*** | ***Grin2a*** | ***Gabra5*** | ***Erk2*** | ***Htr1a*** | ***Grin2b*** | ***Grin2a*** | ***Gabra5*** | ***Mapk1-Erk2*** |
| CS (a) | 0.01 ± 0.008 | 0.16 ± 0.063 | 0.00 ± 0.006 | 0.02 ± 0.008 | 0.00 ± 0.006 | 0.35 ± 0.065 | 0.11 ±0.046 | 0.00 ± 0.003 | 0.04 ± 0.013 | 0.10 ± 0.655 |
| Naïve (b) | 0.06 ± 0.006 | 0.55 ± 0.149 | 0.06 ± 0.003 | 0.33 ± 0.097 | 0.02 ± 0.015 | 0.06 ± 0.002 | 0.40 ± 0.110 | 0.00 ± 0.003 | 0.33 ± 0.098 | 0.04 ± 0.025 |
| Learning(c) | 1.26 ± 0.085^a,b^ | 1.31 ± 0.106^a,b^ | 0.90 ± 0.066^a,b^ | 0.80 ± 0.075^a,b^ | 1.02 ± 0.040^a,b^ | 1.28 ± 0.096^a,b^ | 1.21 ± 0.122^a,b^ | 1.01 ± 0.072^a,b^ | 1.27 ± 0.124^a,b^ | 1.59 ± 0.021^a,b^ |
| Tween^®^ (d) | 1.01 ± 0.110^a,b^ | 1.01 ± 0.120^a,b^ | 0.98 ± 0.044^a,b^ | 1.00 ± 0.045^a,b^ | 1.32 ± 0.030^a,b^ | 1.01 ± 0.105^a,b^ | 1.04 ± 0.204^a,b^ | 1.00 ± 0.010^a,b^ | 1.03 ± 0.195^a,b^ | 1.32 ± 0.001^a,b^ |
| 600 mg.Kg-^1^ Sintocalmy^®^ (e) | 1.21 ± 0.124^a,b^ | 0.98 ± 0.087^a,b^ | 0.28 ± 0.014^a,b^ | 0.93 ± 0.148^a,b^ | 1.24 ± 0.052^a,b^ | 0.91 ± 0.024^a,b^ | 0.93 ± 0.055^a,b^ | 0.27 ± 0.021^a,b^ | 1.15 ± 0.132^a,b^ | 1.26 ± 0.074^a,b^ |
| 0.15 mg.Kg^-1^FfB (f) | 2.30 ± 0.041^a,b,c,d,e^ | 1.00 ± 0.063^a,b^ | 1.08 ± 0.021^a,b,c^ | 1.44 ± 0.087^a,b,c,d,e^ | 1.52 ± 0.145^a,b,c,d,e^ | 1.87 ± 0.023^a,b,c,d,e^ | 1.13 ± 0.086^a,b^ | 1.10 ± 0.041^a,b^ | 1.01 ± 0.057^a,b^ | 2.06 ± 0.010^a,b,c,d,e^ |
| 0.30 mg.Kg^-1^FfB (g) | 2.23 ± 0.016^a,b,c,d,e^ | 0.92 ± 0.072^a,b^ | 1.24 ± 0.056^a,b,c,d^ | 1.57 ± 0.153^a,b,c,d,e^ | 1.96 ± 0.028^a,b,c,d,e^ | 2.31 ± 0.170^a,b,c,d,e^ | 1.04 ± 0.066^a,b^ | 1.11 ± 0.034^a,b^ | 1.13 ± 0.086^a,b^ | 2.56 ± 0.073^a,b,c,d,e^ |
| 0.65mg.Kg^-1^FfB (h) | 2.63 ± 0.093^a,b,c,d,e^ | 0.98 ± 0.040^a,b^ | 1.57 ± 0.076^a,b,c,d^ | 2.05 ± 0.123^a,b,c,d,e^ | 2.01 ± 0.055^a,b,c,d,e^ | 2.47 ± 0.092^a,b,c,d,e^ | 0.94 ± 0.107^a,b^ | 1.28 ± 0.062^a,b^ | 1.34 ± 0.132^a,b^ | 2.50 ± 0.055^a,b,c,d,e^ |

The results are presented as the means (±SEM).

^a^*P*<0.0001 – Comparisons of relative expression for each group x CS group.

^b^*P*<0.0001 – Comparisons of relative expression for each group x Naïve group.

^c^*P*<0.0001– Comparisons of relative expression for each group x learning group.

^d^*P*<0.0001 – Comparisons of relative expression for each group x Tween^®^ group.

^e^*P*<0.0001 – Comparisons of relative expression for each group x 600 mg.Kg-^1^ Sintocalmy^®^ group.
