# Supplementary material for: Effects of a Flavonoid-Rich Fraction on the Acquisition and Extinction of Fear Memory: Pharmacological and Molecular Approaches
Source: Front Behav Neurosci. 2016 Jan 5;9:345. doi: 10.3389/fnbeh.2015.00345 (PMC4700274; doi:10.3389/fnbeh.2015.00345)
Supplement: Supplementary file 5 [file Table4.DOCX]

**Table S4.**Mean suppression ratio (SR) to the CS (tone) at for first trial and three-trial blocks in the retention test, extinction training and extinction retention test for the control groups (saline, (S)-WAY100135 and buspirone) and the treated groups with (S)-WAY100135 + FfB (0.15 mg.Kg^-1^, 0.30 mg.Kg^-1^or 0.65 mg.Kg^-1^).

| **GROUPS** | **TRIALS** | | | | | | | | | | | | | |
| --- | --- | --- | --- | --- | --- | --- | --- | --- | --- | --- | --- | --- | --- | --- |
|  | **Retention test (8^th^ day)** | | | | **Extinction training (9^th^ day)** | | | | | **Extinction Retention test (10^th^ day)** | | | | |
|  | **1** | **2 - 4** | **5-7** | **8-10** | | **1** | **2 - 4** | **5-7** | **8-10** | | **1** | **2 – 4** | **5-7** | **8-10** |
| Saline (a) | 0.75 ± 0.04 | 0.57 ± 0.03^###^ | 0.50 ± 0.02 | 0.53 ± 0.02 | | 0.58 ± 0.02 | 0.57 ± 0.03 | 0.53 ± 0.03 | 0.53 ± 0.03 | | 0.50 ± 0.06 | 0.54 ± 0.03 | 0.50 ± 0.02 | 0.52 ± 0.04 |
| 0.30mg.Kg^-1^(S)-WAY100135 (b) | 0.54 ± 0.03 | 0.57 ± 0.02^###^ | 0.53 ± 0.02 | 0.49 ± 0.02 | | 0.52 ± 0.03 | 0.47 ± 0.03 | 0.48 ± 0.03 | 0.49 ± 0.04 | | 0.46 ± 0.04 | 0.53 ± 0.03 | 0.54 ± 0.03 | 0.45 ± 0.03 |
| 10.0 mg.Kg^-1^Buspirone(c) | 0.74 ± 0.03 | 0.57 ± 0.03 | 0.52 ± 0.02 | 0.55 ± 0.02 | | 0.59 ± 0.07 | 0.59 ± 0.02 | 0.47 ± 0.03 | 0.52 ± 0.02 | | 0.52 ± 0.07 | 0.46 ± 0.03 | 0.49 ± 0.03 | 0.57 ± 0.02 |
| SWAY +0.15 mg.Kg^-1^FfB (d) | 0.61 ± 0 .02^a,c^ | 0.59 ± 0.02 | 0.55 ± 0.02 | 0.48 ± 0.02 | | 0.53 ± 0.03 | 0.48 ± 0.02 | 0.54 ± 0.01 | 0.49 ± 0.03 | | 0.58 ± 0.02 | 0.49 ± 0.03 | 0.49 ± 0.02 | 0.51 ± 0.01 |
| SWAY +0.30 mg.Kg^-1^FfB (e) | 0.61 ± 0.01^a,c^ | 0.50 ± 0.02 | 0.51 ± 0.01 | 0.53 ± 0.03 | | 0.56 ± 0.03 | 0.53 ± 0.03 | 0.54 ± 0.03 | 0.47 ± 0.03 | | 0.56 ± 0.02 | 0.56± 0.01 | 0.53 ± 0.01 | 0.49 ± 0.02 |
| SWAY +0.65 mg.Kg^-1^FfB (f) | 0.62 ± 0.01^a,c^ | 0.54 ± 0.03 | 0.55 ± 0.01 | 0.51 ± 0.01 | | 0.63 ± 0.02 | 0.48± 0.03 | 0.55 ± 0.02 | 0.47 ± 0.03 | | 0.58 ± 0.03 | 0.52 ± 0.03 | 0.50 ± 0.02 | 0.45 ± 0.02 |

The results are presented as the means (±SEM) values.Inter-group and inter-trial comparisons were computed and can be evaluated.

^a^ *P*<0.0001 – Comparison of SR for the first trial for SWAY+ FfB groups x Saline group .

^c^*P*<0.0001 – Comparisons of SR for the first trial for SWAY+ FfB groups x Buspirone group .

^###^ *P*<0.0001 – Comparisons of SR for the first-trial x the first three-trial block (2^th^-4^th^ trial) for each group.
