# Supplementary material for: Effects of a Flavonoid-Rich Fraction on the Acquisition and Extinction of Fear Memory: Pharmacological and Molecular Approaches
Source: Front Behav Neurosci. 2016 Jan 5;9:345. doi: 10.3389/fnbeh.2015.00345 (PMC4700274; doi:10.3389/fnbeh.2015.00345)
Supplement: Supplementary file 6 [file Table5.DOCX]

**Table S5 –** *Htr1a, Grin2b, Grin2a, Gabra5*and *Mapk1/Erk2* expression in the dorsal hippocampus (DH) by qRT-PCR, after the retention test and extinction retention test to the control groups (saline, (S)-WAY100135 and buspirone) and treated with (S)-WAY100135 + FfB (0.15 mg.Kg^-1^, 0.30 mg.Kg^-1^ or 0.65 mg.Kg^-1^).

| **GROUPS** | **Relative expression (ddCt)** | | | | | | | | | | |
| --- | --- | --- | --- | --- | --- | --- | --- | --- | --- | --- | --- |
|  | **Retention test (8^th^ day)** | | | | | **Extinction Retention test (10^th^ day)** | | | | | |
|  | *Htr1a* | ***Grin2b*** | ***Grin2a*** | ***Gabra5*** | ***Erk2*** | | *Htr1a* | ***Grin2b*** | ***Grin2a*** | ***Gabra5*** | ***Erk2*** |
| Saline (a) | 1.01 ± 0.107 | 1.00 ± 0.066 | 1.00 ± 0.103 | 1.00 ± 0.065 | 1.01 ± 0.136 | | 1.10 ± 0.110 | 1.08 ± 0.294 | 1.00 ± 0.066 | 1.03 ± 0.195 | 1.00 ± 0.07 |
| 0.30mg.Kg^-1^(S)-WAY100135 (b) | 0.44 ± 0.041^a,c^ | 0.81 ± 0.063 | 0.15 ± 0.030^a,c^ | 1.55 ± 0.187 | 1.08 ± 0.053 | | 0.94 ± 0.217^a,b,d,e,f^ | 4.44 ± 0.714^a,d,e,f^ | 0.07 ± 0.005^a^ | 0.55 ± 0.106 | 0.85 ± 0.04 |
| 10.0 mg.Kg^-1^Buspirone(c) | 1.29 ± 0.081 | 0.47 ± 0.119^a,b^ | 0.93 ± 0.080 | 1.22 ± 0.223 | 0.85 ± 0.069 | | 21.52 ± 2.36 | 2.69 ± 0.198^a,d,e,f^ | 0.03 ± 0.000 ^a^ | 0.65 ± 0.004 | 1.05 ± 0.09 |
| SWAY +0.15 mg.Kg^-1^FfB (d) | 0.25 ± 0.042^a,c^ | 0.40 ± 0.014^a,b^ | 0.04 ± 0.014^a,c^ | 1.01 ± 0.171 | 0.83 ± 0.053 | | 1.29 ± 0.112 | 0.80 ± 0.050 | 1.67 ± 0.113^a,b,c^ | 0.41 ± 0.092 | 1.16 ± 0.10 |
| SWAY +0.30 mg.Kg^-1^FfB (e) | 0.38 ± 0.040^a,c^ | 0.58± 0.003^a,b^ | 0.28 ± 0.003^a,c^ | 1.12 ± 0.066 | 0.85 ± 0.032 | | 1.04 ± 0.086 | 0.83 ± 0.038 | 2.22 ± 0.085^a,b,c^ | 0.59 ± 0.046 | 1.12 ± 0.05 |
| SWAY +0.65 mg.Kg^-1^FfB (f) | 0.42 ± 0.051^a,c^ | 0.63 ± 0.012^a,b^ | 0.33 ± 0.012^a,c^ | 0.93 ± 0.027 | 1.10 ± 0.008 | | 1.55 ± 0.057 | 0.91 ± 0.083 | 2.55 ± 0.011^a,b,c^ | 0.57 ± 0.037 | 1.19 ± 0.03 |

The results are presented as means (±SEM) values.

^a^*P*<0.0001 – Comparisons of relative expression for each group x saline group.

^b^*P*<0.0001 – Comparisonsof relative expression for each group x 0.30mg.Kg^-1^(S)-WAY100135 group.

^c^*P*<0.0001 – Comparisons of relative expression for each group x buspironegroup.

^d^*P*<0.0001 – Comparisonsof relative expression for each group x SWAY +0.15 mg.Kg^-1^FfB group.

^e^*P*<0.0001 – Comparisons of relative expression for each group x SWAY +0.30 mg.Kg^-1^FfB group.

^f^*P*<0.0001 – Comparisons of relative expression for each group x SWAY +0.65 mg.Kg^-1^FfB group
