# Supplementary material for: Effects of a Flavonoid-Rich Fraction on the Acquisition and Extinction of Fear Memory: Pharmacological and Molecular Approaches
Source: Front Behav Neurosci. 2016 Jan 5;9:345. doi: 10.3389/fnbeh.2015.00345 (PMC4700274; doi:10.3389/fnbeh.2015.00345)
Supplement: Supplementary file 7 [file Table6.DOCX]

**Table S6.**Mean suppression ratio (SR) to the CS (tone) at first trial and three-trial blocks in the retention test, extinction training and extinction retention test to the control groups (saline, Ro25-6981 and NMDA) and treated with Ro25-6981 + FfB (0.15 mg.Kg^-1^, 0.30 mg.Kg^-1^or 0.65 mg.Kg^-1^.

| **GROUPS** | **TRIALS** | | | | | | | | | | | | |
| --- | --- | --- | --- | --- | --- | --- | --- | --- | --- | --- | --- | --- | --- |
|  | **Retention test (8^th^ day)** | | | | **Extinction training (9^th^ day)** | | | | **Extinction Retention test (10^th^ day)** | | | | |
|  | **1** | **2 - 4** | **5-7** | **8-10** | **1** | **2 - 4** | **5-7** | **8-10** | **1** | **2 – 4** | **5-7** | **8-10** |  |
| Saline (a) | 0.75 ± 0.04^b^ | 0.57 ± 0.03^###^ | 0.50 ± 0.02 | 0.53 ± 0.02 | 0.58 ± 0.02 | 0.57 ± 0.03 | 0.53 ± 0.03 | 0.53 ± 0.03 | 0.50 ± 0.06 | 0.54 ± 0.03 | 0.50 ± 0.02 | 0.52 ± 0.04 | |
| 3.0 mg.Kg^-1^ Ro25-6981 (b) | 0.39 ± 0.02 | 0.53 ± 0.01 | 0.53 ± 0.02 | 0.49 ± 0.05 | 0.63 ± 0.05 | 0.56 ± 0.02 | 0.54 ±0.03 | 0.56 ± 0.03 | 0.54 ± 0.07 | 0.54 ± 0.03 | 0.47 ± 0.03 | 0.52 ± 0.03 | |
| 10 mg.Kg^-1^NMDA (c) | 0.68 ± 0.07 ^b^ | 0.52 ± 0.03^###^ | 0.57 ± 0.03 | 0.51 ± 0.02 | 0.56 ± 0.06 | 0.56 ± 0.02 | 0.49 ± 0.03 | 0.48 ± 0.03 | 0.58 ± 0.04 | 0.50 ± 0.04 | 0.54 ± 0.04 | 0.49 ± 0.02 | |
| Ro+ 0.15mg.Kg^-1^FfB (d) | 0.67 ± 0.05 ^b^ | 0.53 ± 0.03^###^ | 0.56 ± 0.02 | 0.51 ± 0.01 | 0.60 ± 0.02 | 0.52 ± 0.02 | 0.54 ± 0.01 | 0.49 ± 0.03 | 0.60 ± 0.04 ^a^ | 0.51 ± 0.02^###^ | 0.59 ± 0.02 | 0.56 ± 0.03 | |
| Ro+0.30mg.Kg^-1^FfB FfB (e) | 0.73 ± 0.02 ^b^ | 0.55 ± 0.01^###^ | 0.53 ± 0.03 | 0.49 ± 0.01 | 0.64 ± 0.01 | 0.58 ± 0.01 | 0.55 ± 0.01 | 0.48 ± 0.02 | 0.59 ± 0.02 ^a^ | 0.57 ± 0.01 | 0.51 ± 0.02 | 0.50 ± 0.01 | |
| Ro+ 0.65mg.Kg^-1^FfB (f) | 0.78 ± 0.02 ^b^ | 0.58 ± 0.02^###^ | 0.54 ± 0.02 | 0.52± 0.01 | 0.67 ± 0.04 | 0.60 ± 0.01 | 0.56 ± 0.04 | 0.54 ± 0.01 | 0.65 ± 0.05 ^a^ | 0.50 ± 0.01^###^ | 0.52 ± 0.02 | 0.48 ± 0.02 | |

The results are presented as means (±SEM) values. Comparisons inter-group and inter-trial were realized and can be evaluated.

^a^*P*<0.0001 – Comparisons of SR for the first trial for Ro+ FfB groups x saline group .

**^b^***P*<0.0001-Comparisonsof SR for the first trial for each group x 3.0 mg.Kg^-1^ Ro25-6981 group .

^###^ *P*<0.0001-Comparisonsof SR for the first-trial x the first three-trial block (2^th^-4^th^ trial) for each group.
