# Supplementary material for: Effects of a Flavonoid-Rich Fraction on the Acquisition and Extinction of Fear Memory: Pharmacological and Molecular Approaches
Source: Front Behav Neurosci. 2016 Jan 5;9:345. doi: 10.3389/fnbeh.2015.00345 (PMC4700274; doi:10.3389/fnbeh.2015.00345)
Supplement: Supplementary file 8 [file Table7.DOCX]

**Table S7-** *Htr1a, Grin2b, Grin2a, Gabra5*and *Mapk1/Erk2* expression in the dorsal hippocampus (DH) by qRT-PCR, after the retention test and extinction retention test to the control groups (saline, Ro25-6981 and NMDA) and treated with Ro25-6981 + FfB (0.15 mg.Kg^1^, 0.30 mg.Kg^-1^ or 0.65 mg.Kg^-1^.

| **GROUPS** | **Relative expression (ddCt)** | | | | | | | | | | |
| --- | --- | --- | --- | --- | --- | --- | --- | --- | --- | --- | --- |
|  | **Retention test (8^th^ day)** | | | | | **Extinction Retention test (10^th^ day)** | | | | | |
|  | ***Htr1a*** | ***Grin2b*** | ***Grin2a*** | ***Gabra5*** | ***Erk2*** | | ***Hhtr1a*** | ***Grin2b*** | ***Grin2a*** | ***Gabra5*** | ***Erk2*** |
| Saline (a) | 1.01 ± 0.107 | 1.00 ± 0.066 | 1.00 ± 0.103 | 1.00 ± 0.065 | 1.01 ± 0.136 | | 1.10 ± 0.110 | 1.08 ± 0.294 | 1.00 ± 0.066 | 1.03 ± 0.195 | 1.69 ± 0.61 |
| 3.0 mg.Kg^-1^ Ro25-6981 (b) | 0.02 ± 0.016 ^a^ | 0.54 ± 0.071 | 1.52 ± 0.142 | 0.08 ± 0.032 ^a, c^ | 0.05 ± 0.020 ^a^ | | 17.6 ±1.513^a,c^ | 1.52 ± 0.141 | 1.44 ± 0.164 | 0.88 ± 0.126 | 2.61 ± 0.080 |
| 10 mg.Kg^-1^NMDA (c) | 0.08 ± 0.066 ^a^ | 3.02 ± 0.497^a,b,d,e,f^ | 1.26 ± 0.068 | 0.43 ± 0.131 ^a^ | 2.41 ± 0.068^a,b^ | | 0.17 ± 0.061 | 2.98 ± 0.112^a,b^ | 1.23 ± 0.169 | 0.76 ± 0.096 | 2.21 ± 0.51 |
| Ro+ 0.15mg.Kg^-1^FfB (d) | 0.52 ± 0.062^a,b,c^ | 0.61 ± 0.049 | 3.40 ± 0.221^a,b,c^ | 0.51 ± 0.092^a,b^ | 3.02 ± 0.412^a,b,c^ | | 23.38 ± 1.46^a,c^ | 1.90 ± 0.286 ^a^ | 1.93 ± 0.044^a,b,C^ | 0.74 ± 0.020 | 3.46 ± 0.132^a,B,c^ |
| Ro+0.30mg.Kg^-1^FfB FfB (e) | 0.44 ± 0.049^a,b,c^ | 0.58 ± 0.056 | 4.46 ± 0.188^a,b,c^ | 0.44 ± 0.107^a,b^ | 4.41 ± 0.174^a,b,c^ | | 23.05 ± 0.40^a,c^ | 1.77 ± 0.367 ^a^ | 2.11 ± 0.16^,b,c^ | 0.82 ± 0.058 | 3.55 ± 0.242^a,B,c^ |
| Ro+ 0.65mg.Kg^-1^FfB (f) | 0.52 ± 0.028^a,b,c^ | 0.52 ± 0.048 | 4.62 ± 0.15^a,b,c^ | 0.84 ± 0.100 ^b^ | 4.68 ± 0.021^a,b,c^ | | 26.78 ± 0.79^a,c^ | 2.35 ± 0.475 ^a^ | 2.19 ± 0.104^a,b,c^ | 0.73 ± 0.008 | 3.80 ± 0.016^a,B,c^ |

The results are presented as means (±SEM) values.

^a^*P*<0.0001 – Comparisons of relative expression for each group x saline group.

^b^*P*<0.0001 – Comparisons of relative expression for each group x 3.0 mg.Kg^-1^Ro25-6981 group.

^c^*P*<0.0001 – Comparisons of relative expression for each group x 10 mg.Kg^-1^NMDAgroup.

^B^*P*<0.001 – Comparisons of relative expression for all groups Ro+ FfBx 3.0 mg.Kg^-1^Ro25-6981 group.

^C^ *P*<0.05 – Comparisons of relative expression for Ro+ 0.15mg.Kg^-1^FfBx10 mg.Kg^-1^NMDA group.
