# Supplementary material for: Effects of a Flavonoid-Rich Fraction on the Acquisition and Extinction of Fear Memory: Pharmacological and Molecular Approaches
Source: Front Behav Neurosci. 2016 Jan 5;9:345. doi: 10.3389/fnbeh.2015.00345 (PMC4700274; doi:10.3389/fnbeh.2015.00345)
Supplement: Supplementary file 9 [file Table8.DOCX]

**Table S8 -** Mean suppression ratio (SR) to the CS (tone) at first trial and three-trial blocks in the retention test, extinction training and extinction retention test to the control groups (saline, picrotoxin and diazepam) and treated with picrotoxin+ FfB (0.15 mg.Kg^-1^, 0.30 mg.Kg^-1^or 0.65 mg.Kg^-1^.

| **GROUPS** | **TRIALS** | | | | | | | | | | | |
| --- | --- | --- | --- | --- | --- | --- | --- | --- | --- | --- | --- | --- |
|  | **Retention test (8^th^ day)** | | | | **Extinction training (9^th^ day)** | | | | **Extinction Retention test (10^th^ day)** | | | |
|  | **1** | **2 - 3** | **5-7** | **8-10** | **1** | **2 - 3** | **5-7** | **8-10** | **1** | **2 – 3** | **5-7** | **8-10** |
| Saline (a) | 0.75 ± 0.04 ^c^ | 0.57 ± 0.03^###^ | 0.50 ± 0.02 | 0.53 ± 0.02 | 0.58 ± 0.02 | 0.57 ± 0.03 | 0.53 ± 0.03 | 0.53 ± 0.03 | 0.50 ± 0.06 | 0.54 ± 0.03 | 0.50 ± 0.02 | 0.52 ± 0.04 |
| 0.75 mg.Kg-1 Picrotoxin (b) | 0.66 ± 0.05 ^c^ | 0.53 ± 0.03^###^ | 0.49 ± 0.02 | 0.48 ± 0.02 | 0.60 ± 0.04 | 0.51 ± 0.04 | 0.53 ± 0.04 | 0.58 ± 0.04 | 0.46 ± 0.07 | 0.50 ± 0.03 | 0.51 ± 0.02 | 0.45 ± 0.02 |
| 4.0 mg.Kg^-1^ Diazepam (c) | 0.37 ± 0.03 | 0.48 ± 0.03 | 0.51 ± 0.02 | 0.56 ± 0.04 | 0.46 ± 0.08 | 0.54 ± 0.02 | 0.45 ± 0.03 | 0.52 ± 0.03 | 0.50 ± 0.04 | 0.48 ± 0.02 | 0.53 ± 0.02 | 0.53 ± 0.04 |
| Picro+0.15 mg.Kg^-1^FfB (d) | 0.75 ± 0.04 ^c^ | 0.54 ± 0.02^###^ | 0.57 ± 0.02 | 0.49 ± 0.02 | 0.71 ± 0.01^a,b^ | 0.54 ± 0.02^###^ | 0.52 ± 0.02 | 0.51 ± 0.02 | 0.66 ± 0.02^a,b^ | 0.51 ± 0.02^###^ | 0.48 ± 0.03 | 0.46 ± 0.02 |
| Picro+0.30 mg.Kg^-1^FfB (e) | 0.67 ± 0.04 ^c^ | 0.56 ± 0.02^###^ | 0.55 ± 0.03 | 0.51 ± 0.03 | 0.67 ± 0.01^a,b^ | 0.55 ± 0.03^###^ | 0.49 ± 0.03 | 0.49 ± 0.02 | 0.62 ± 0.02^a,b^ | 0.50 ± 0.01^###^ | 0.49 ± 0.01 | 0.52 ± 0.02 |
| Picro+0.65 mg.Kg^-1^FfB (f) | 0.69± 0.03 ^c^ | 0.55 ± 0.02^###^ | 0.50 ± 0.02 | 0.51 ± 0.02 | 0.74 ± 0.02^a,b^ | 0.52 ± 0.04^###^ | 0.51 ± 0.04 | 0.53 ± 0.03 | 0.66 ± 0.02^a,b^ | 0.51 ± 0.03^###^ | 0.50 ± 0.02 | 0.46 ± 0.02 |

The results are presented as means (±SEM) values. Comparisons inter-group and inter-trial were realized and can be evaluated.

^a^*P*<0.0001 -Comparisons of SR for the first trial for picro+ FfB groups x saline group .

^b^*P*<0.0001 – Comparisons of SR for the first trial for picro+ FfB groups x 0.75 mg.Kg^-1^picrotoxingroup .

**^c^***P*<0.0001 – Comparisons of SR for the first trial for all groups x 4.0 mg.Kg^-1^diazepam group .

^###^ *P*<0.0001 – Comparisons of SR for the first-trial x the first three-trial block (2^th^-4^th^ trial) for each group.
