# Supplementary material for: Effects of a Flavonoid-Rich Fraction on the Acquisition and Extinction of Fear Memory: Pharmacological and Molecular Approaches
Source: Front Behav Neurosci. 2016 Jan 5;9:345. doi: 10.3389/fnbeh.2015.00345 (PMC4700274; doi:10.3389/fnbeh.2015.00345)
Supplement: Supplementary file 10 [file Table9.DOCX]

**Table S9 -** *Htr1a, Grin2b, Grin2a, Gabra5* and *Mapk1/Erk2* expression in the dorsal hippocampus (DH) by qRT-PCR, after the retention test and extinction retention test to the control groups (saline, picrotoxin and diazepam) and treated with picrotoxin+ FfB (0.15 mg.Kg^1^, 0.30 mg.Kg^-1^ and0.65 mg.Kg^-1^.

| **GROUPS** | **Relative expression (ddCt)** | | | | | | | | | |
| --- | --- | --- | --- | --- | --- | --- | --- | --- | --- | --- |
|  | **Retention test (8^th^ day)** | | | | | **Extinction Retention test (10^th^ day)** | | | | |
|  | ***Htr1a*** | ***Grin2b*** | ***Grin2a*** | ***Gabra5*** | ***Erk2*** | ***Htr1a*** | ***Grin2b*** | ***Grin2a*** | ***Gabra5*** | ***Erk2*** |
| Saline (a) | 1.01 ± 0.107 | 1.00 ± 0.066 | 1.00 ± 0.103 | 1.00 ± 0.065 | 1.01 ± 0.136 | 1.10 ± 0.110 | 1.08 ± 0.294 | 1.00 ± 0.066 | 1.03 ± 0.195 | 1.03 ± 0.195 |
| 0.75 mg.Kg^-1^Picrotoxin (b) | 1.15 ± 0.064 | 0.58 ± 0.144 ^a^ | 0.93 ± 0.045 | 1.49 ± 0.121 | 1.44 ± 0.075 ^a^ | 30.34 ± 8.36^a,c,d,e,f^ | 2.65 ± 0.379^a,c,d,e,f^ | 1.02 ± 0.901 | 2.16 ± 0.422^a,c,d,e,f^ | 1.08 ± 0.079 |
| 4.0 mg.Kg^-1^ Diazepam (c) | 0.60 ± 0.078^a,b^ | 3.10 ± 0.011^a,b,d,e,f^ | 0.18 ± 0.054^a,b,d,e,f^ | 1.09 ± 0.183^a,b,d,e,f^ | 0.49 ± 0.006^a,b^ | 5.27 ± 1.301^a,d,e,f^ | 0.32 ± 0.036 | 0.18 ± 0.054^a,b^ | 0.19 ± 0.048 ^a,^ | 0.96 ± 0.083 |
| Picro+0.15 mg.Kg^-1^FfB (d) | 0.98 ± 0.058 ^c^ | 0.55 ± 0.038 ^a^ | 0.72 ± 0.046 | 0.71 ± 0.017 | 1.52 ± 0.046^a,b,c^ | 1.36 ± 0.305 ^a^ | 0.36 ± 0.071 | 1.52 ± 0.101^a,b,c^ | 0.72 ± 0.005 | 1.94 ± 0.074^a,b,c^ |
| Picro+0.30 mg.Kg^-1^FfB (e) | 1.50 ± 0.034^a,b,c,d^ | 0.49 ± 0.050 ^a^ | 1.07 ± 0.200 | 0.84 ± 0.023 | 2.43 ± 0.099^a,b,c,d^ | 2.75 ± 0.131 ^a,^ | 0.40 ± 0.073 | 1.73 ± 0.026^a,b,c^ | 0.51 ± 0.005 | 2.42 ± 0.121^a,b,c,d^ |
| Picro+0.65 mg.Kg^-1^FfB (f) | 1.60 ± 0.046^a,b,c,d^ | 0.50 ± 0.026^a,b,c,d^ | 1.03 ± 0.058 | 0.99 ± 0.058 | 2.38 ± 0.066^a,b,c,d^ | 3.66 ± 0.073 ^a,^ | 0.40 ± 0.038 | 1.70 ± 0.063^a,b,c^ | 0.95 ± 0.072 | 3.19 ± 0.125^a,b,c,d^ |

The results are presented as means (±SEM) values.

^a^*P*<0.0001 Comparisons of relative expression for each group x saline group.

^b^*P*<0.0001 Comparisons of relative expression for each group x .0.75 mg.Kg^-1^picrotoxingroup.

^c^*P*<0.0001 Comparisons of relative expression for each group x 4.0 mg.Kg^-1^diazepamgroup.

^d^*P*<0.0001 Comparisons of relative expression for each group x picro+0.15 mg.Kg^-1^FfBgroup.

^e^*P*<0.0001 Comparisons of relative expression for each group x picro+0.30 mg.Kg^-1^FfBgroup.

^f^*P*<0.0001 Comparisons of relative expression for each group x picro+0.65 mg.Kg^-1^FfBgroup
